# Photoinduced 1,2,3,4-tetrahydropyridine ring conversions

**DOI:** 10.3762/bjoc.11.234

**Published:** 2015-11-11

**Authors:** Baiba Turovska, Henning Lund, Viesturs Lūsis, Anna Lielpētere, Edvards Liepiņš, Sergejs Beljakovs, Inguna Goba, Jānis Stradiņš

**Affiliations:** 1 Physical-organic Chemistry Laboratory, Latvian Institute of Organic Synthesis, 21 Aizkraukles Str., Riga LV-1006, Latvia; 2 Department of Organic Chemistry, Aarhus University, Langelandsgade 140, DK 8000 Aarhus, Denmark

**Keywords:** heterocyclic hydroperoxide, oxaziridine, photoinduced electron transfer, pyrrolidine, tetrahydropyridine

## Abstract

Stable heterocyclic hydroperoxide can be easily prepared as a product of fast oxidation of a 1,2,3,4-tetrahydropyridine by ^3^O_2_ if the solution is exposed to sunlight. The driving force for the photoinduced electron transfer is calculated from electrochemical and spectroscopic data. The outcome of the reaction depends on the light intensity and the concentration of O_2_. In the solid state the heterocyclic hydroperoxide is stable; in solution it is involved in further reactions.

## Introduction

Increased attention has been paid to the chemistry of cyclic organic peroxides since it was found that naturally occurring representatives of this group possess biological activity, particular antimalarial [1–2]. Significantly less attention has been paid to organic aromatic or heterocyclic hydroperoxides, probably due to their low thermal stability and high reactivity.

Stable organic hydroperoxides were isolated in the early 1950s as products of autoxidation as well as catalytic oxygenation of indoles and tetrahydrocarbazoles [3–6]. In 1944 hydroperoxides were first obtained by H. Hock [7] and R. Udris [8–10] as the catalytic oxidation products of cumene. Heterocyclic hydroperoxides have been less represented, although some of these constitute the best choice for selective oxidations even in nature. When the peroxy functional group is placed near to electronegative groups, the oxidizing capability of hydroperoxides can be increased. This effect was particularly observed on heterocyclic systems.

## Results and Discussion

Photosensitized aerobic oxidative aromatization [11–14] of Hantzsch 1,4-dihydropyridines has been extensively investigated, in the same time little attention has been paid to the corresponding reactions of tetrahydropyridines.

**Figure 1:**
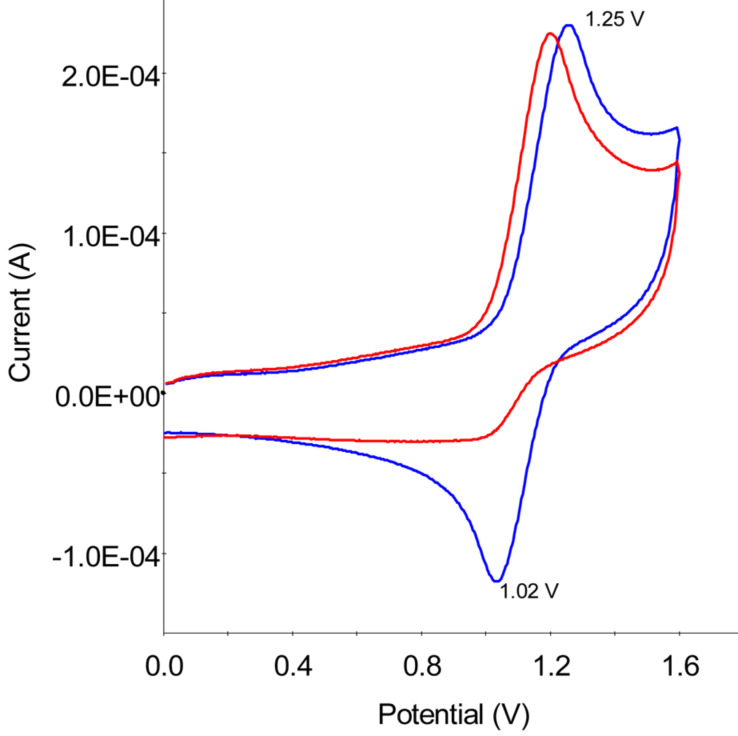
Electrochemical oxidation of **1** in deareated (blue) and O_2_ saturated (red) solutions of CH_2_Cl_2_/0.1 M TBAPF_6_, *c* = 5 × 10^−4^ M.

During the investigation of the electrochemical oxidation mechanism of tetrahydropyridine **1** [15], an extremely high sensitivity of the formed cation radicals towards traces of dioxygen was observed. In deaereated aprotic solvents **1** undergoes a reversible one-electron single-step oxidation [16] (+1.00 V in MeCN or +1.25 V in CH_2_Cl_2_) while the reversibility of the anodic process disappears immediately after the argon flow through/over the solution has been stopped (Figure 1).

**Figure 2:**
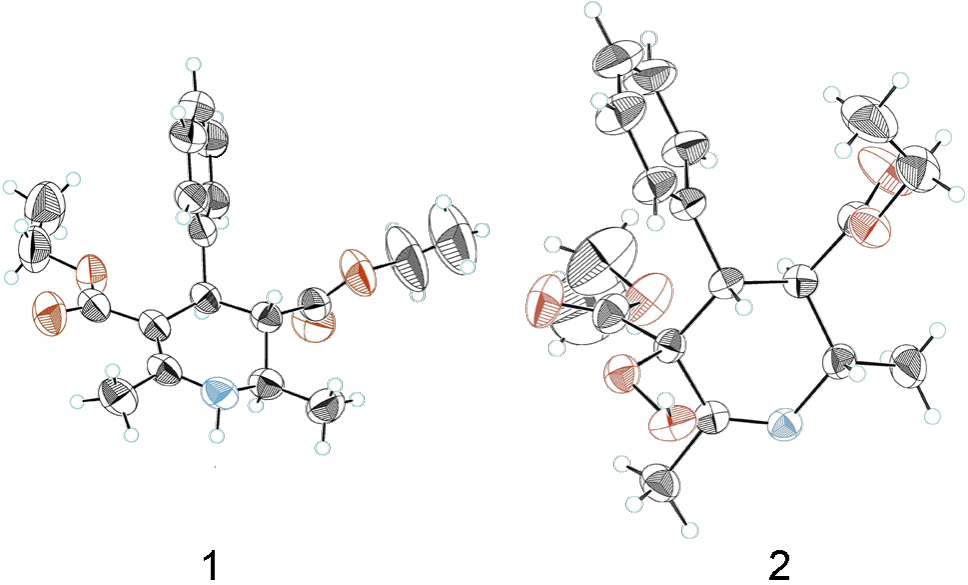
The X-ray structures of compounds **1** and **2**.

Moreover, it was found that tetrahydropyridine **1** (Figure 2) reacts with dioxygen if the solution is exposed to intense sunlight. In order to study this reaction, the solution of **1** (0.5 g, 1.51 mmol) was left under an irradiation of intense sunlight and continuously purged with dioxygen (~9.1 mmol) [17–18] in chloroform (25 mL). Crystals suitable for X-ray analysis were obtained after evaporation of the chloroform by a stream of dioxygen; elemental analysis, ^1^H, ^13^C NMR spectra and X-ray analysis confirmed the structure of hydroperoxide **2** (Figure 2) as the only product of the reaction. Hydroperoxide **2** is a colourless crystalline compound which is stable in the solid state but unstable in solution.

The oxidation of tetrahydropyridine **1** is photoinduced; it does not take place in the dark and it depends on the intensity of the light. No reaction could be initiated by light in a deareated solution.

The reaction of dioxygen (^3^O_2_) having a triplet ground state with tetrahydropyridine **1** having a singlet ground state is spin forbidden. On the other hand, the electron transfer from the organic compound to ^3^O_2_ resulting in the formation of a radical cation of the organic donor and the radical anion of O_2_ (O_2_^−•^) is spin permitted [19], however, it is not thermodynamically favourable.

The driving force for the photoinduced electron transfer can be calculated from electrochemical and spectroscopic measurements made in the same solvent according to Weller’s approximation [20]


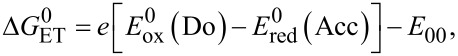


where 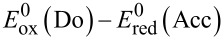 is the difference between the first oxidation potential of the donor and the first reduction potential of the acceptor;


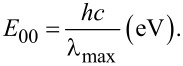


In CH_2_Cl_2_ the reduction potential of O_2_ is −1.18 V and *E*^0^ of **1** is +1.14 V.


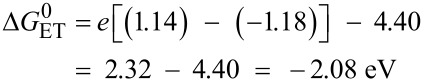


The reaction becomes thermodynamically favourable if the organic molecule is excited to a singlet state. The oxidation potential of excited molecules is shifted to negative values (Δ*E* = ~2 V) compared to their ground state [21–23]. For example the oxidation potential of 9,10-dihydro-10-methylacridine is +0.80 V [23], which is shifted to −3.10 V in the singlet excited state [23].

**Figure 3:**
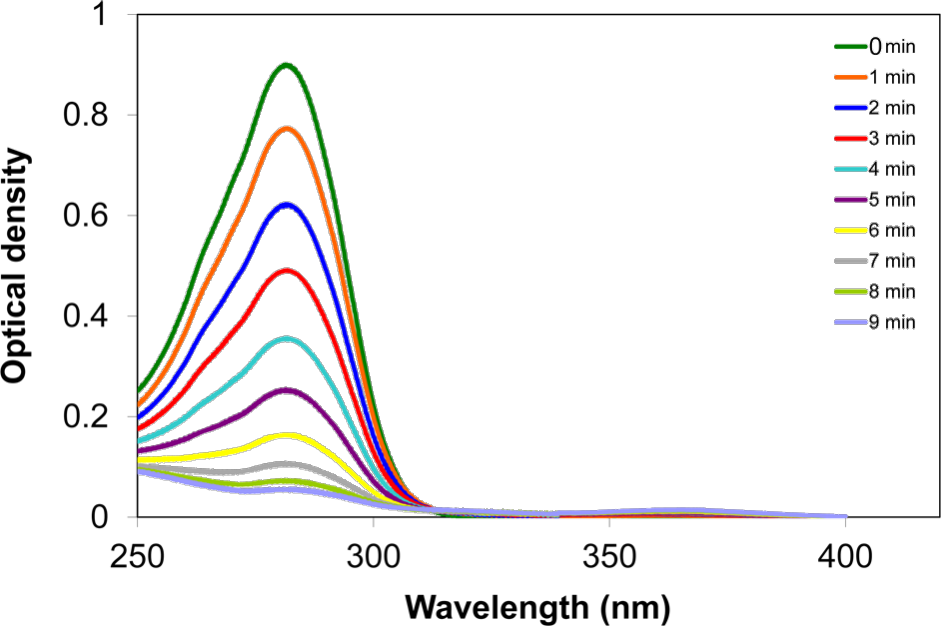
Decrease of the UV absorption band of compound **1** under irradiation (254 nm) in air-saturated CHCl_3_, *c* = 5 × 10^−5^ M.

The recorded UV–vis spectrum of **1** (Figure 3) has only one absorption band at λ_max_ = 282 nm in the range of 250–900 nm which corresponds to the spin allowed S→S* transition (ε = 19000).

If a series of UV spectra is recorded after subsequent periods of 1 min irradiation of the sample with an external UV lamp (254 nm, 8 W), the absorption band decreases and no new band appears in the range of 250–900 nm (Figure 3). If the irradiation of the sample is stopped for 1 min, the reaction halts, and the recorded UV spectrum exactly repeats the previous one.

It might be suggested that the initiation step, excitation of **1** at 254 nm, leading to the formation of the singlet state of 1*, is followed by single-electron transfer from 1* to ^3^O_2_ generating **1**^+•^ and O_2_^− •^ in solution. Such reactions between strong nucleophiles and strong electrophiles, especially the annihilation reactions between ion radicals, have not been studied extensively, probably due to the difficulties of generating anion and cation radicals simultaneously [24–25].

The reactivity of superoxide is widely studied by chemists and biochemists. It has been shown electrochemically that O_2_^− •^ is a product of the reversible one-electron reduction of dioxygen in aprotic solvents [26–27]. Superoxide O_2_^−•^ is a highly reactive molecule [26,28] and it acts as a strong Brönsted base removing a proton from substrates to an extent equivalent to that of the conjugate base with a p*K*_a_ value of approximately 23 in water [29–30].

**Scheme 1:**
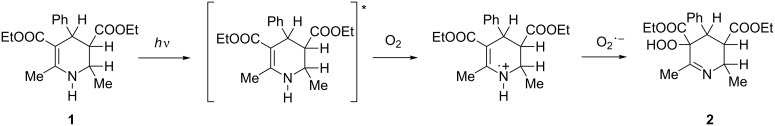
Photoinduced reaction of **1** in O_2_ saturated CHCl_3_ under irradiation by intensive sunlight.

A number of weakly acidic organic compounds are deprotonated efficiently in the presence of superoxide including Hantzsch 1,4-dihydropyridines [31–32]. Cation radicals have increased acidity comparing to the parent molecules from which they are derived by oxidation [33–35]. Consequently, their deprotonation proceeds more efficiently. Photooxygenation of **1** can be described as shown in Scheme 1.

**Scheme 2:**
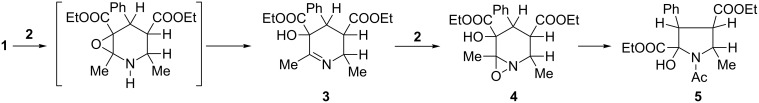
Heterocycle transformations of **1** in air saturated CHCl_3_ solutions.

In an air saturated CHCl_3_ solution, the concentration of dioxygen is about 2 mmol/L [17–18] and if it becomes comparable with the concentration of **1**, the reaction takes a different course (Scheme 2).

It has been suggested that peracids attack the carbon–carbon double bond in enamines to give the corresponding epoxides, however, in most cases they have not been isolated [36].

**Scheme 3:**
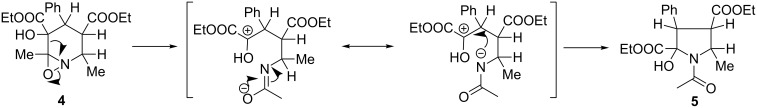
Proposed mechanism of conversion of oxaziridine **4** to **5**.

**Figure 4:**
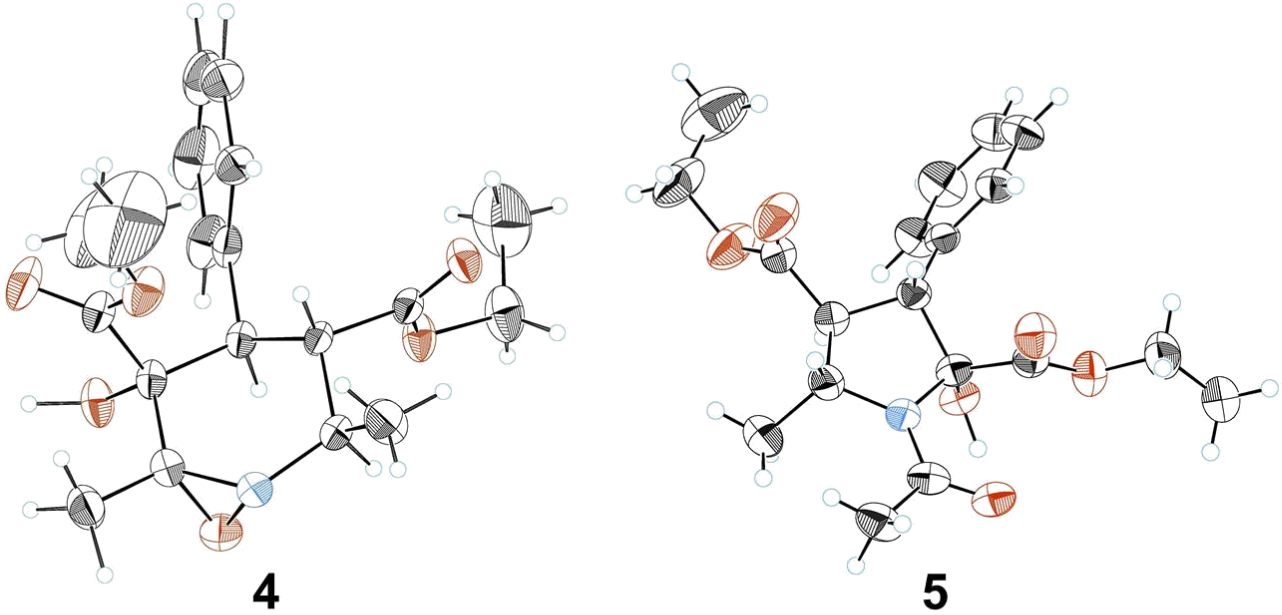
The X-ray structures of compounds **4** and **5**.

The hydroperoxide **2** reacts in the same way. Fission of the epoxide ring may be induced by the base itself producing **3**. Attack of a second molecule of hydroperoxide **2** on the imine group of **3** oxidizes it to oxaziridine intermediate **4** (Figure 4), which then undergoes a slow nucleophilic ring fission followed by cyclization (Scheme 3) to give **5** (Figure 4).

The reaction depicted in Scheme 2 proceeds as long as the hydroperoxide **2** is present in the solution, and the crude product contains the mixture of **3**, **4** and **5**. The same product mixture was obtained if the solution of **1** is treated with concentrated H_2_O_2_ or Fenton reagent.

The reaction starts even if the irradiation wavelengths only partially cover the absorption band of **1**. It explains also the observed fact that during the summer as well as under bright laboratory light the photooxygenation of tetrahydropyridine **1** in the presence of dioxygen proceeds spontaneously in CHCl_3_, CH_2_Cl_2_ or CH_3_CN solutions. Although much of the light emitted by the sun in UV below 300 nm is absorbed by ordinary glass, the transmitted intensity of the sunlight in summer at 300 nm is sufficient to initiate the photooxygenation of **1**.

Direct reactions of dioxygen with organic substrates in the absence of a catalyst are usually slow, unless the substrate is a particularly good reducing agent. Excited state species are easier to oxidize than the corresponding ground state species.

The reaction between photochemically generated radical cations and radical anions by electron transfer from photoexcited electron donor to electron acceptors is often reversible [37] thus reproducing the reactant pair without the formation of a chemical bond. As the dioxygen has a large reorganization energy for electron transfer [38–39], the reverse reaction may be retarded, promoting to the subsequent heterocycle conversions.

## Conclusion

Several synthetic routes have been explored towards chiral hydroperoxides as they have been utilized successfully in a variety of asymmetric oxidations. We have found a sunlight activated reaction of 1,2,3,4-tetrahydropiridine with dioxygen (^3^O_2_) producing stable heterocyclic hydroperoxide in excellent yield. The reaction has significant advantages as it uses cost-free reagents: light and dioxygen. The same reaction demonstrated the oxidizing capacity of the hydroperoxide when its concentration is less or comparable with the tetrahydropyridine leading to another versatile intermediate in organic synthesis – oxaziridine – as the inherent strain of the ring and the relatively weak N–O bond makes it unusually reactive.

## Supporting Information

Synthesis and characterization of all products, copies of ^1^H and ^13^C NMR spectra of newly synthesized products.

File 1 Experimental and analytical data.
