# Supplementary material for: Photoinduced 1,2,3,4-tetrahydropyridine ring conversions
Source: Beilstein J Org Chem. 2015 Nov 11;11:2166–70. doi: 10.3762/bjoc.11.234 (PMC4660982; doi:10.3762/bjoc.11.234)
Supplement: File 1 — Experimental and analytical data. [file Beilstein_J_Org_Chem-11-2166-s001.pdf]

Supporting Information

for

Photoinduced 1,2,3,4-tetrahydropyridine ring conversions

Baiba Turovska*[1], Henning Lund[2], Viesturs Lūsis[1], Anna Lielpētere[1], Edvards Liepiņš[1], Sergejs Beljakovs[1], Inguna Goba[1] and Jānis Stradiņš[1]

Address: [1]Physical-organic Chemistry Laboratory, Latvian Institute of Organic Synthesis, 21 Aizkraukles Str., Riga LV-1006, Latvia and [2]Department of Organic Chemistry, Aarhus University, Langelandsgade 140, DK 8000 Aarhus, Denmark

Email: Baiba Turovska* - turovska@osi.lv

* Corresponding author

Experimental and analytical data

Methods and instruments

The reaction products were analysed by ^{1}H and ^{13}C NMR spectra registered on a Bruker DMX 500 spectrometer. The progress of the photochemical reaction of **1** was monitored by ^{1}H NMR spectra on a Bruker WH 90 spectrometer.

Cyclic voltammetry was recorded using Advanced Electrochemical System PARSTAT 2273.

UV spectra were recorded on Camspec M501 Single Beam Scanning UV/Visible Spectrophotometer.

Infrared spectra were recorded on a Perkin-Elmer 580B spectrometer.

GC-MS spectra were obtained by Hewlett-Packard Model GC 6890 gas chromatograph coupled with mass selective detector.

Elemental analyses were performed at EA 1106 (Carlo Erba Instruments).

Melting points were obtained on a BÜCHI 535.

X-ray crystallographic analysis were done on Nonius Kappa CCD.

Experimental section

3,5-Diethoxycarbonyl-2,6-dimethyl-4-phenyl-1,2,3,4-tetrahydropyridine (**1**) was synthesized from the corresponding 1,4-dihydropyridine according to a general procedure[1].

^{1}H NMR (500 MHz, $CDCl_3$, δ): 0.71 (t, *J*= 7.0 Hz, 3-$COOCH_2\mathit{CH_3}$, 3H), 1.03 (t, *J*= 7.0 Hz, 5-$COOCH_2\mathit{CH_3}$, 3H), 1.11 (d, *J*= 7.0 Hz, 2-CH_3, 3H), 2.30 (d, *J*= 1.0 Hz, 6-CH_3, 3H), 2.42 (t, *J*= 10.0 Hz, 3-H, 1H), 3.52 (m, *J*= 10.0 & 7.0 & 1.0 Hz, 2-H, 1H), 3.71 (dq, *J*= 7.0 & 1.2 Hz, 3-$COO\mathit{CH_2}CH_3$, 2H), 3.94 (broad s, NH, 1H), 3.98 (q, *J*= 7.0 Hz, 5-$COO\mathit{CH_2}CH_3$, 2H), 4.06 (broad d, *J*= 10.0 Hz, 4-H, 1H), 7.08 (m, ArH, 5H) ppm.

Mp 144-146 ºC.

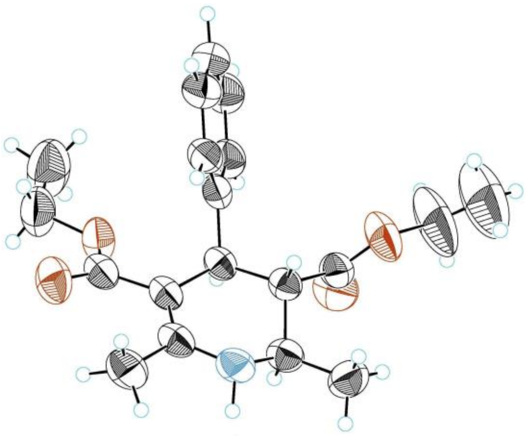

1

X-ray structure (CCDC 667862)

[1] Rosentreter, U. *Synthesis* **1985**, 2, 210.

I Oxidation of 1 in solutions saturated by dioxygen

3,5-Diethoxycarbonyl-2,6-dimethyl-3-hydroperoxy-4-phenyl-3,4,5,6-tetrahydropyridine (2)

0.12080 g (0.36 mmol) of 3,5-diethoxycarbonyl-2,6-dimethyl-4-phenyl-1,2,3,4-tetrahydropyridine (**1**) was dissolved in 20 mL $CHCl_3$. Oxygen was intensivly blew through the solution, therefore the volumetric flask of bigger volume (50 mL) was prefered. The solution was exposed to direct sunlight. After 35 min $CHCl_3$ was evaporated due to the continuous flow of dioxygen and crystals of hydroperoxy **2** suitable for X-ray diffraction analysis were obtained.

^{1}H NMR (500 MHz, $CDCl_3$, δ): 0.97 (t, *J*= 7.0 Hz, 5-$COOCH_2$*CH_3*, 3H), 1.07 (t, *J* = 7.0 Hz, 3-$COOCH_2$*CH_3*, 3H), 1.45 (d, *J*= 7.0 Hz, 6-CH_3, 3H), 2.09 (d, *J*= 2.1 Hz, 2-CH_3, 3H), 3.41 (dd, *J*= 13.3 & 9.8 Hz, 5-H, 1H), 3.85 (m, *J*= 9.8 & 7.0 & 2.1 Hz, 6-H, 1H), 3.95 (AB m, *J*= 7.0 Hz, 5-COO*CH_2*CH_3, 2H), 3.98 (q, *J*= 7.0 Hz, 3-COO*CH_2*CH_3, 2H), 4.06 (d, *J*= 13.3 Hz, 4-H, 1H), 7.20 (m, ArH, 5H), 9.43 (bs, OOH, 1H) ppm.

^{13}C NMR (50 MHz, $CDCl_3$, δ): 13.67 (5-$COOCH_2$*CH_3*), 13.83 (3-$COOCH_2$*CH_3*), 21.60 (6-CH_3), 21.66 (2-CH_3), 43.71 (4-C), 49.01 (5-C), 56.67 (6-C), 60.64 (5-COO*CH_2*CH_3), 62.00 (3-COO*CH_2*CH_3), 87.85 (3-C), 127.58 & 127.99 & 128.86 & 135.92 (ArC), 163.78 (2-C), 166.28 (3-*CO*OCH_2CH_3), 172.63 (5-*CO*OCH_2CH_3) ppm.

Anal. Calcd for $C_{19}H_{25}NO_6$: C, 62.80; H, 6.93; N, 3.85. Found: C, 62.87; H, 6.87; N, 3.86. Mp 126-127 ºC.

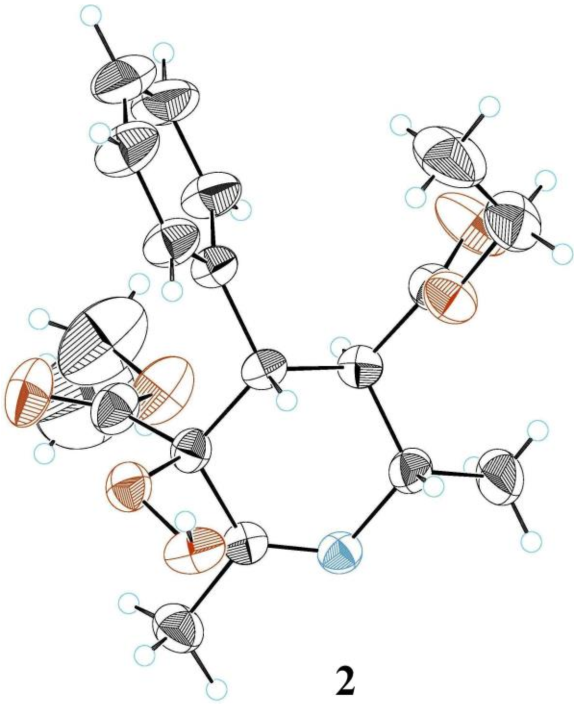

2

X-ray structure (CCDC 667861)

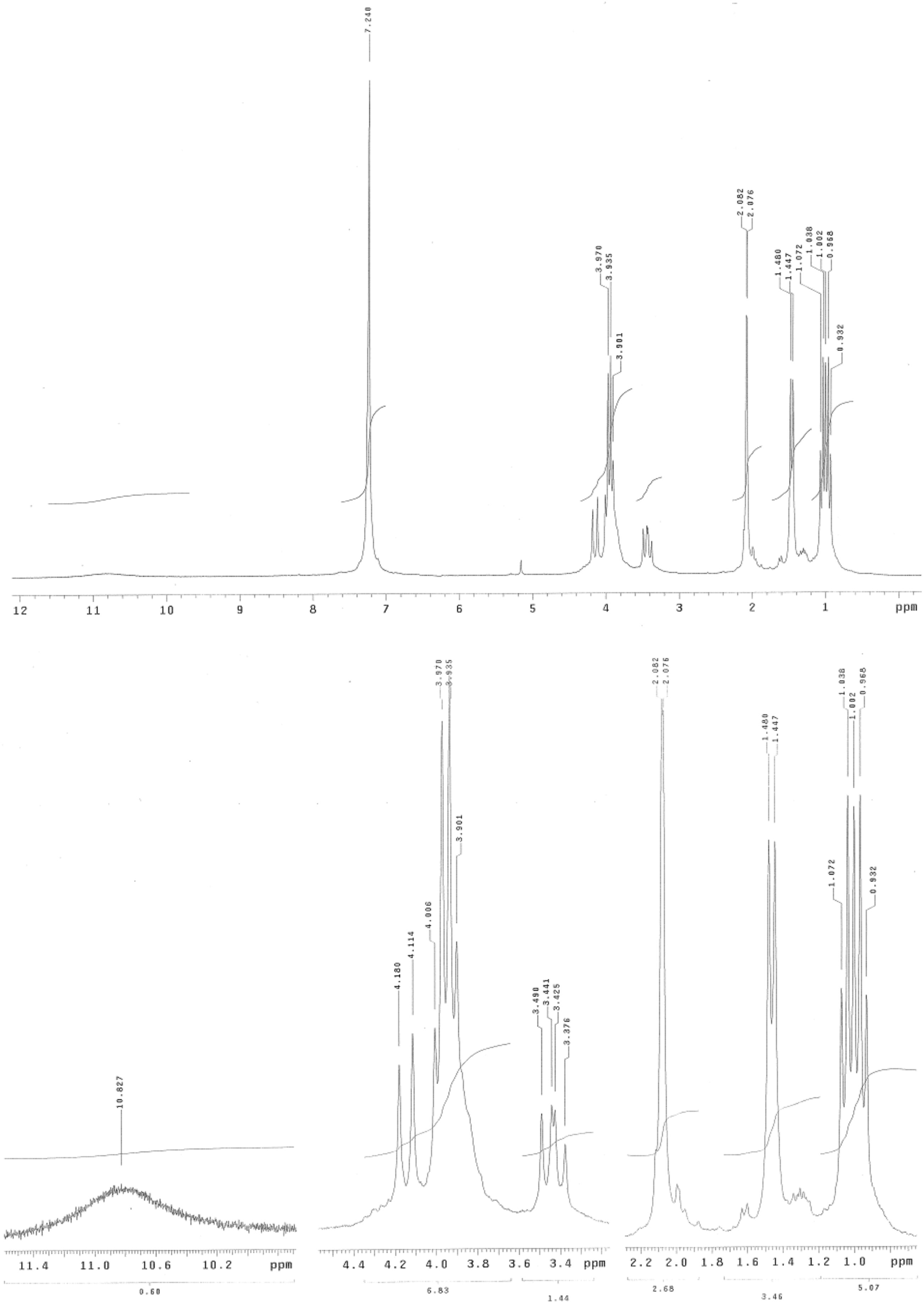

^{1}H NMR spectra in $CDCl_3$ of **2**

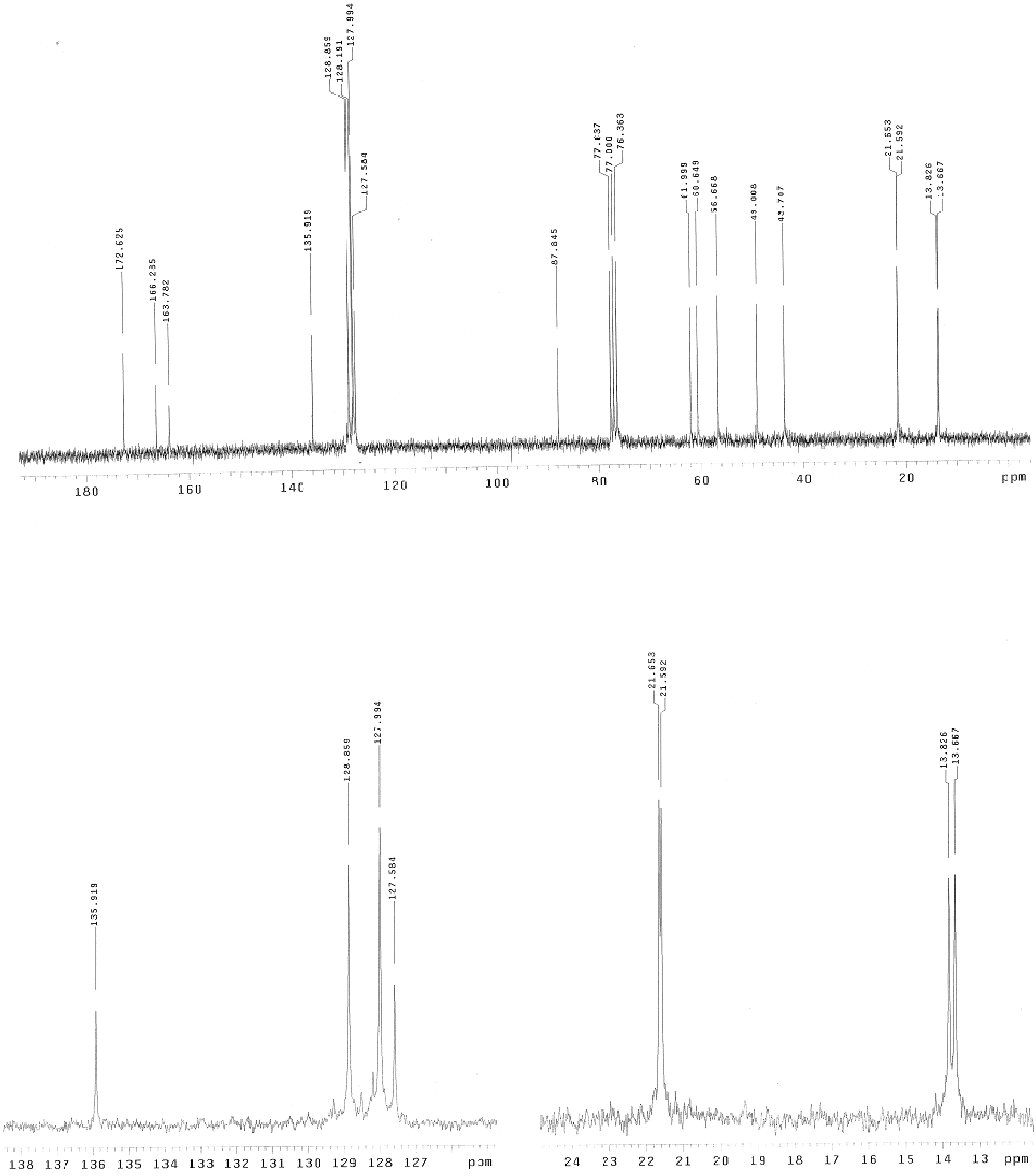

^{13}C NMR specta in $CDCl_3$ of **2**

II Oxidation of 1 in air saturated solutions

1.13955 g (3.44 mmol) of 3,5-diethoxycarbonyl-2,6-dimethyl-4-phenyl-1,2,3,4-tetrahydropyridine (**1**) was dissolved in 50 mL $CDCl_3$. The solution was stirred in a opened glass flask exposed to the direct sunlight. The reaction was controlled by ^{1}H NMR spectroscopy. After completion of the reaction (*t* ~ 1 h) the solvent was evaporated, the crude product was charged on a silicia gel (0.035–0.07 mm Acros Organics) column (h = 28 cm, d = 2 cm) and eluted using chloroform/petrolium ether/acetone (9:7:1); 10 mL fractions were collected. Combined fractions 12–18 following after the dead volume (110 mL) contained 0.29315 g (0.81 mmol) of pure 3,5-diethoxycarbonyl-2,6-dimethyl-3-hydroxy-4-phenyl-1,2-epoxypiperidine (**4**) which was concentrated in vacuum to obtain a white solid. Single crystals suitable for X-ray diffraction analysis were obtained by slow evaporation of a hexane solution.

Fractions 21–30 that contained a mixture of 3,5-diethoxycarbonyl-2,6-dimethyl-3-hydroxy-4-phenyl-3,4,5,6-tetrahydropyridine (**3**) and *N*-acetyl-2,4-diethoxycarbonyl-2-hydroxy-4-methyl-3-phenylpyrolidine (**5**) were combined and after solvent removal (rotary evaporator) a pale yellow oil (0.8377 g) was obtained. Pure **5** was isolated from the oil by crystalization from diethyl ether/hexane. The white precipitate 0.44530 g (1.23 mmol) was removed by filtration. Crystals for X-ray diffraction analysis were grown from hexane.

The filtrate containing a mixture of 3,5-diethoxycarbonyl-2,6-dimethyl-3-hydroxy-4-phenyl-3,4,5,6-tetrahydropyridine (**3**) and *N*-acetyl-2,4-diethoxycarbonyl-2-hydroxy-4-methyl-3-phenylpyrolidine (**5**) was concentrated in vacuum and analysed by 1D and 2D NMR spectra.

The isolation of the products is the same if ethyl acetate/hexane (1:1) was used as an eluent (**4** R_f 0.48; **3** R_f 0.16; **5** R_f 0.15) for column chromatography.

3,5-Diethoxycarbonyl-2,6-dimethyl-3-hydroxy-4-phenyl-3,4,5,6-tetrahydropyridine (3)

^{1}H NMR (500 MHz, $CDCl_3$, δ): 0.94 (t, *J*= 7.0 Hz, 5-$COOCH_2$*CH_3,* 3H), 1.36 (t, *J* = 7.0 Hz, 3-$COOCH_2$*CH_3*, 3H), 1.46 (d, *J*= 7.3 Hz, 6-CH_3, 3H), 1.95 (d, *J*= 2.1 Hz, 2-CH_3, 3H), 3.43 (dd, *J*= 13.4 & 9.5 Hz, 5-H, 1H), 3.51 (d, *J*= 13.4 Hz, 4-H, 1H), 3.78 (s, OH, 1H), 3.94 (m, *J*= 9.5 & 7.3 & 2.1 Hz, 6-H, lH), 3.96 (AB *m,* *J*= 7.0 Hz, 5-COO*CH_2*CH_3, 2H), 4.20 (AB m, *J* = 7.0 Hz, 3-COO*CH_2*CH_3, 2H), 7.20 (m, ArH, 5H) ppm.

^{13}C NMR (125 MHz, $CDCl_3$, δ): 13.6 (5-$COOCH_2$*CH_3*), 13.8 (3-$COOCH_2CH_3$), 21.7 (6-CH_3), 21.9 (2-CH_3), 49.3 (5-C), 49.8 (4-C), 56.8 (6-C), 60.4 (5-COO*CH_2*CH_3), 63.2 (3-COO*CH_2*CH_3), 77.3 (3-C), 129.0 & 129.5 & 135 (ArC), 162.8 (2-C), 170.8 (3-*CO*OCH_2CH_3), 171.9 (5-*CO*OCH_2CH_3) ppm.

IR (dry film, cm^{-1}): 3490 (br), 1730 (s), 1670 (m).

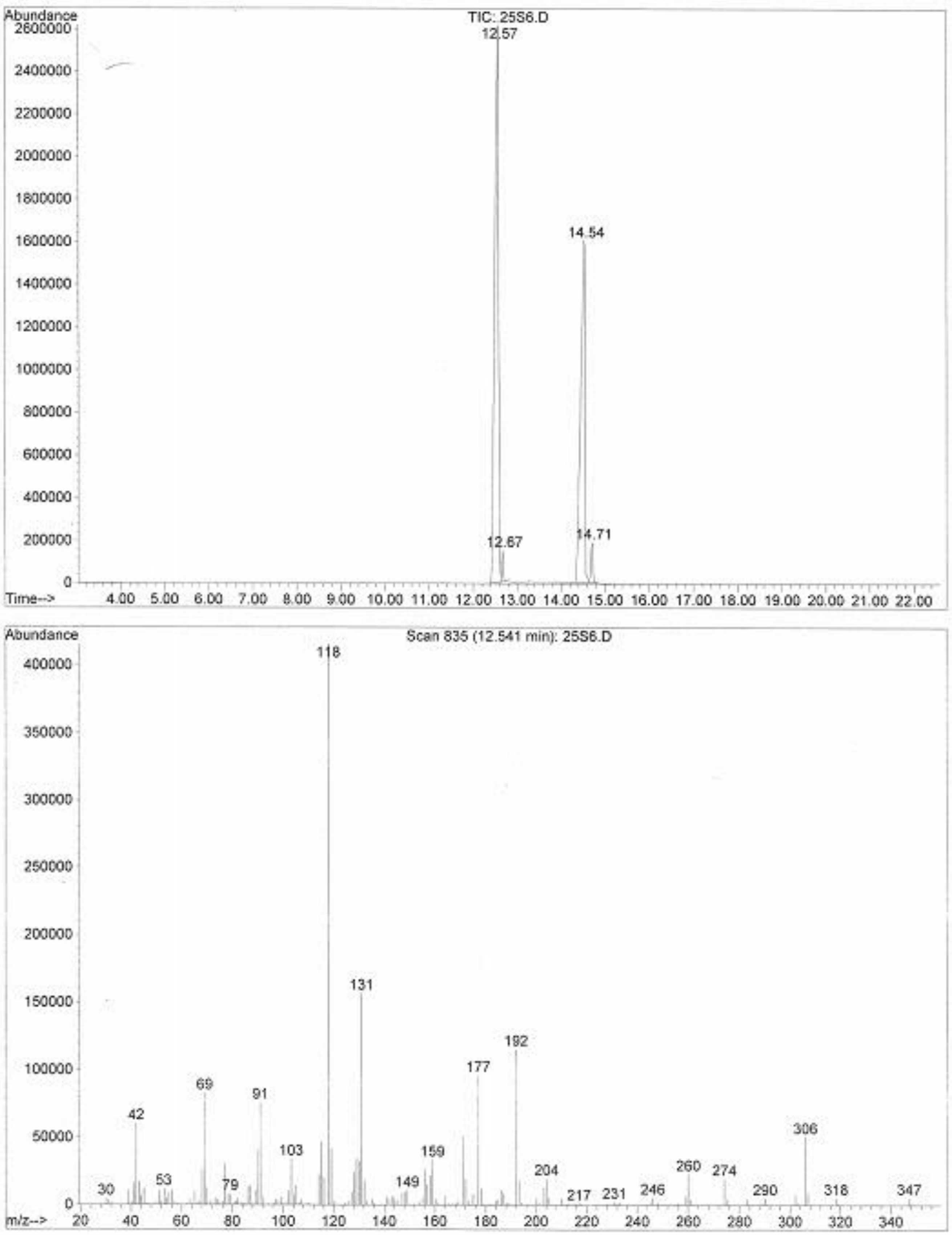

GC–MS spectrum of **3** and **5**

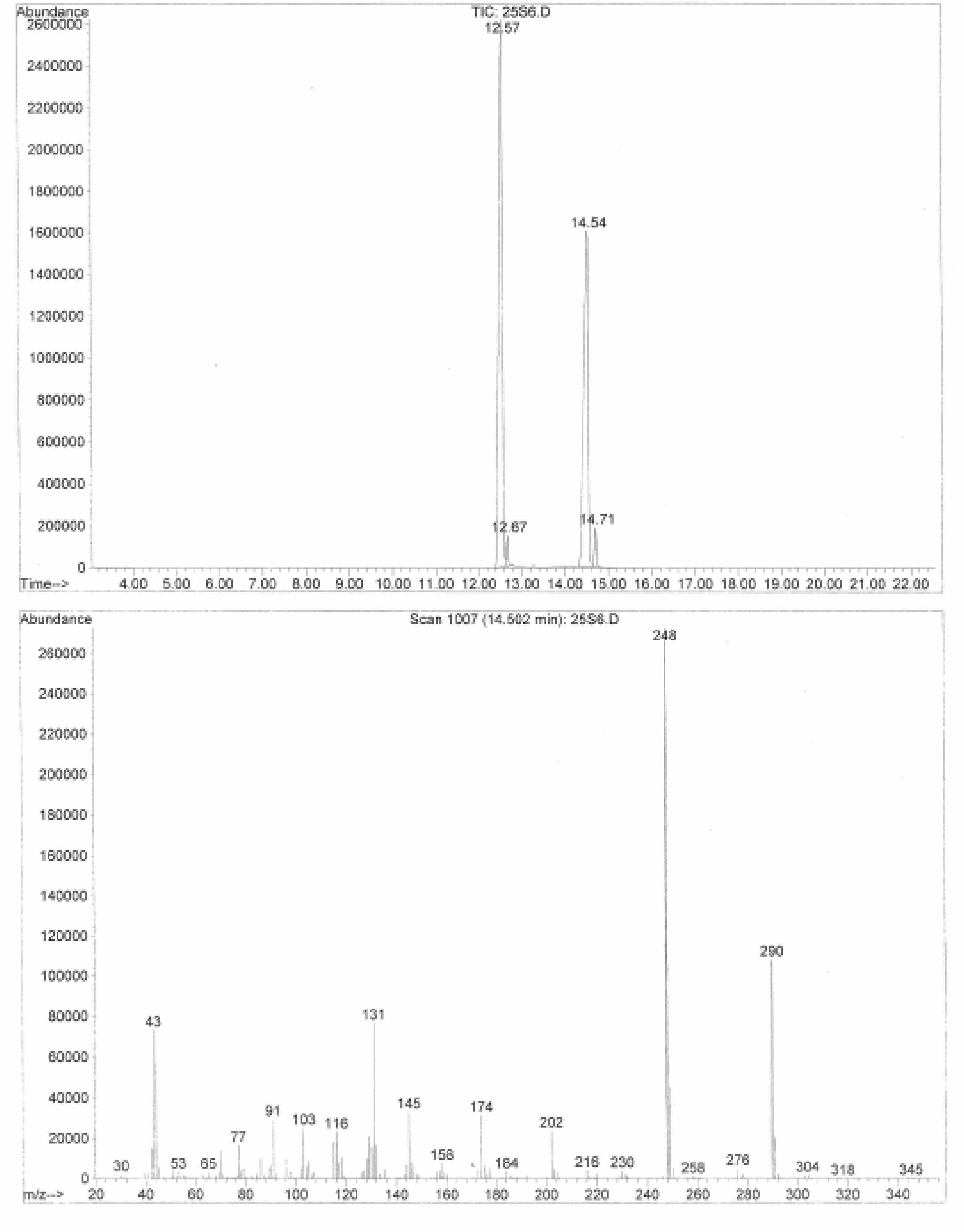

GC–MS spectrum of **3** and **5**

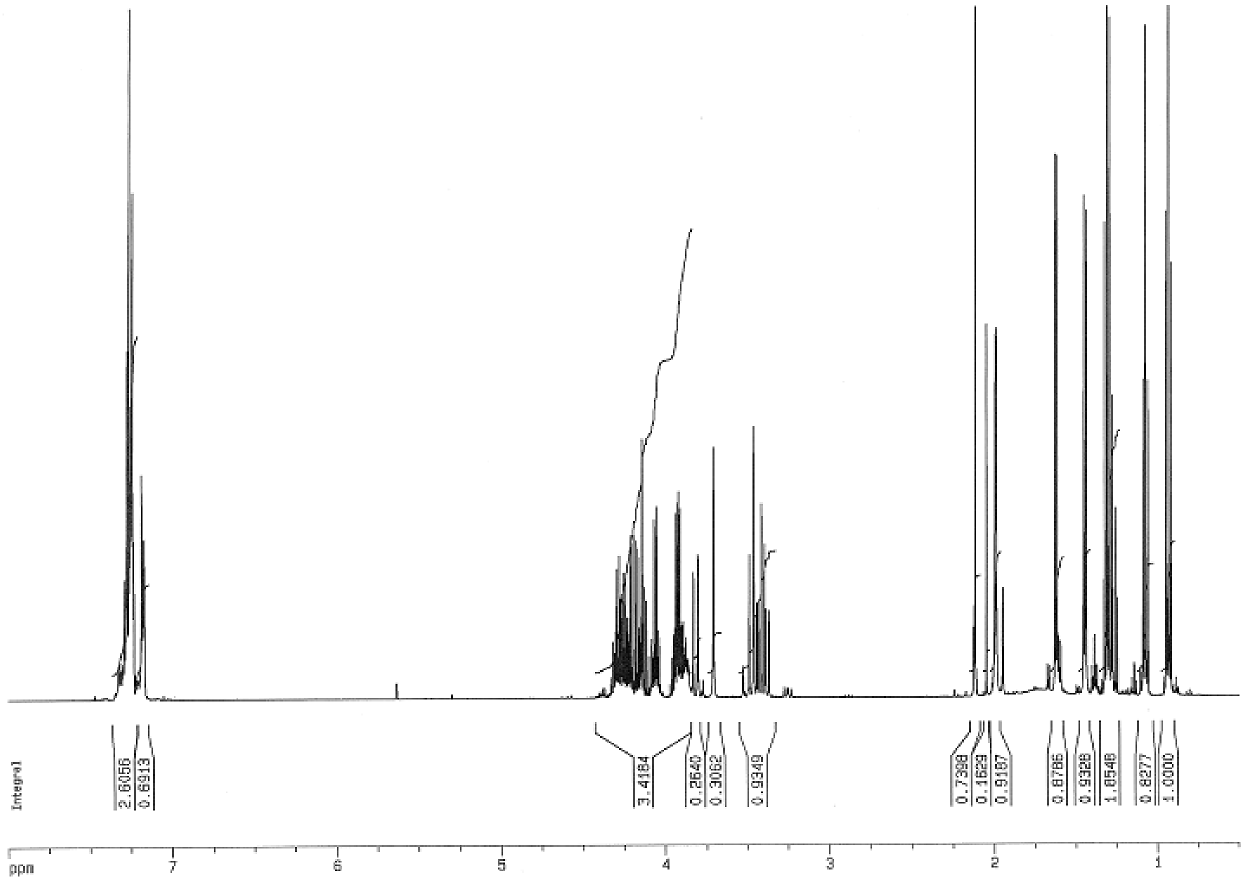

Integral
2.6056
0.6913
3.4184
0.2640
0.3062
0.9349
0.7398
0.1629
0.9187
0.8786
0.9328
1.8548
0.8277
1.0000
ppm
7
6
5
4
3
2
1

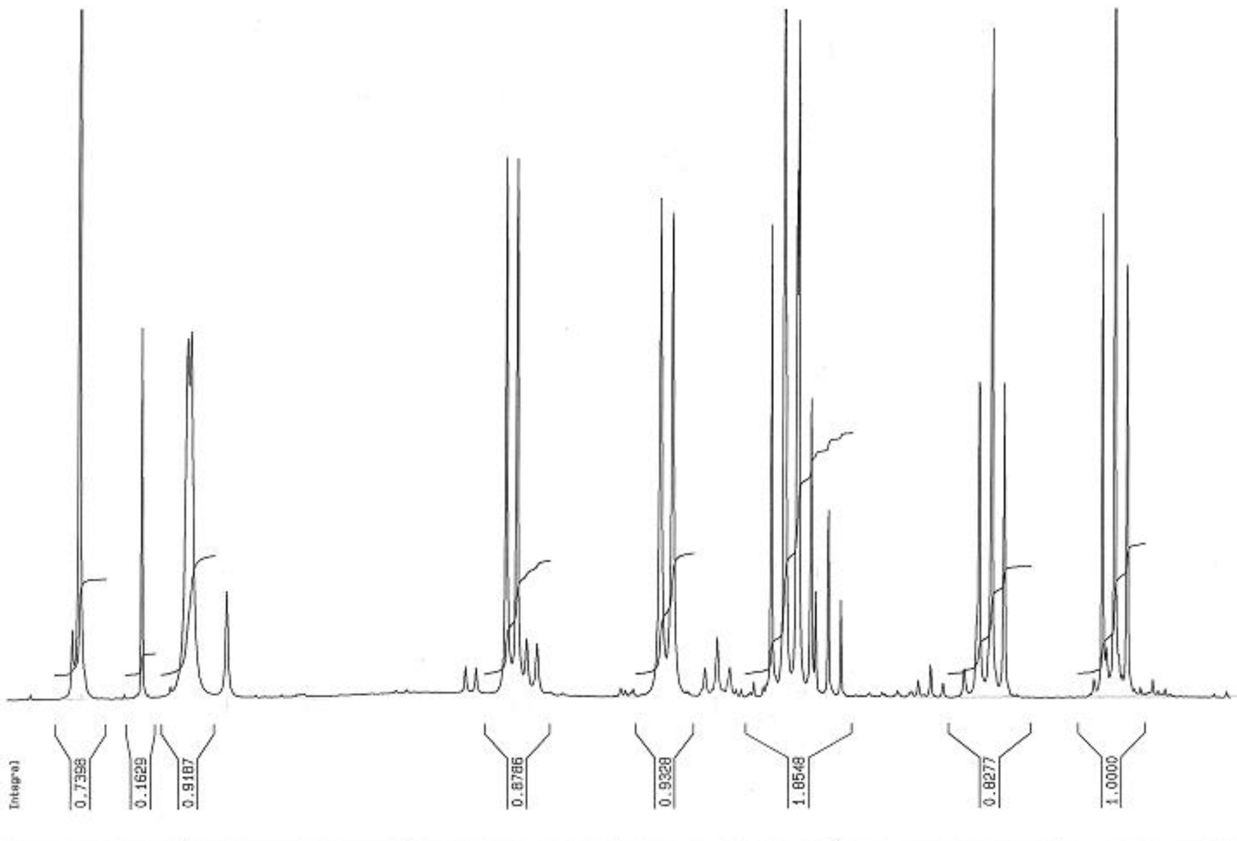

Integral
0.7398
0.1629
0.9187
0.8786
0.9328
1.8548
0.8277
1.0000
ppm
2.00
1.75
1.50
1.25
1.00

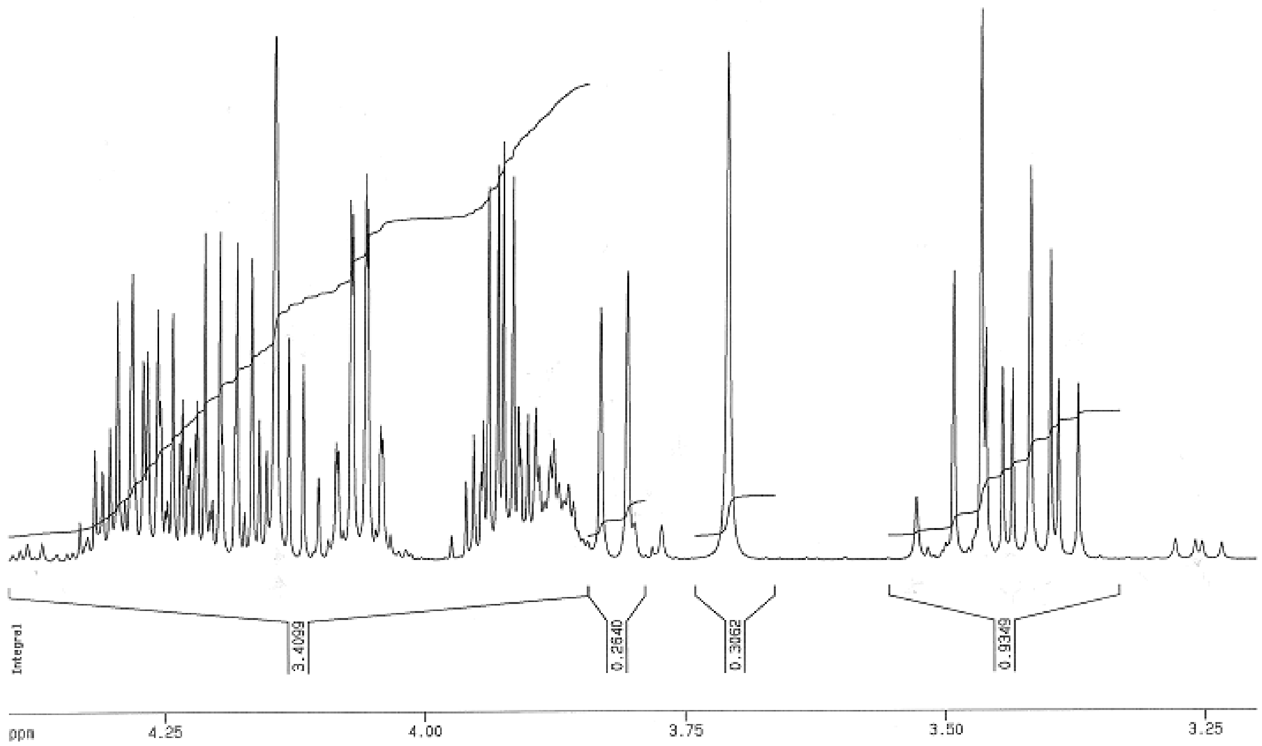

^{1}H NMR spectra in $CDCl_3$ of **3** and **5**

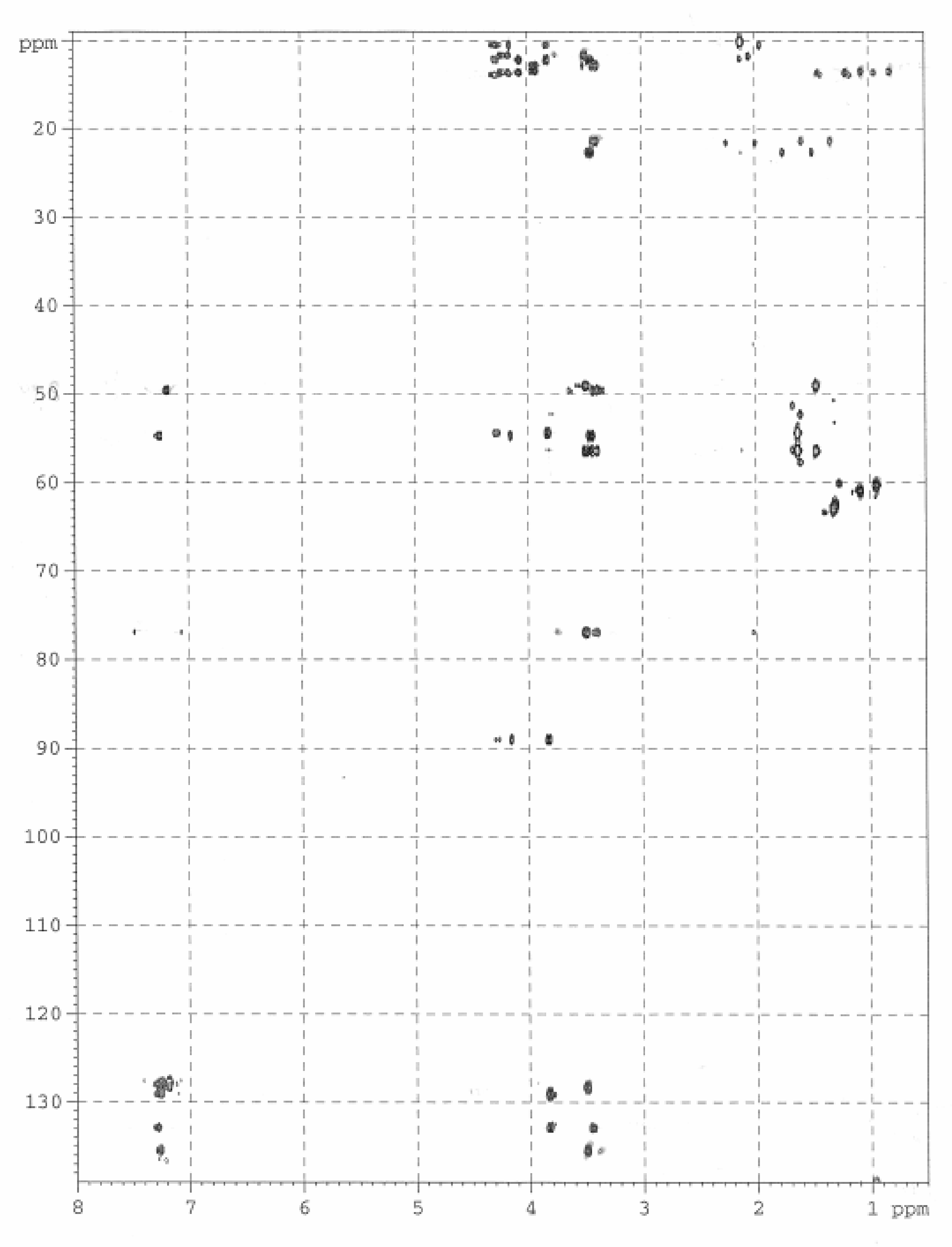
ppm
20
30
40
50
60
70
80
90
100
110
120
130
8
7
6
5
4
3
2
1 ppm

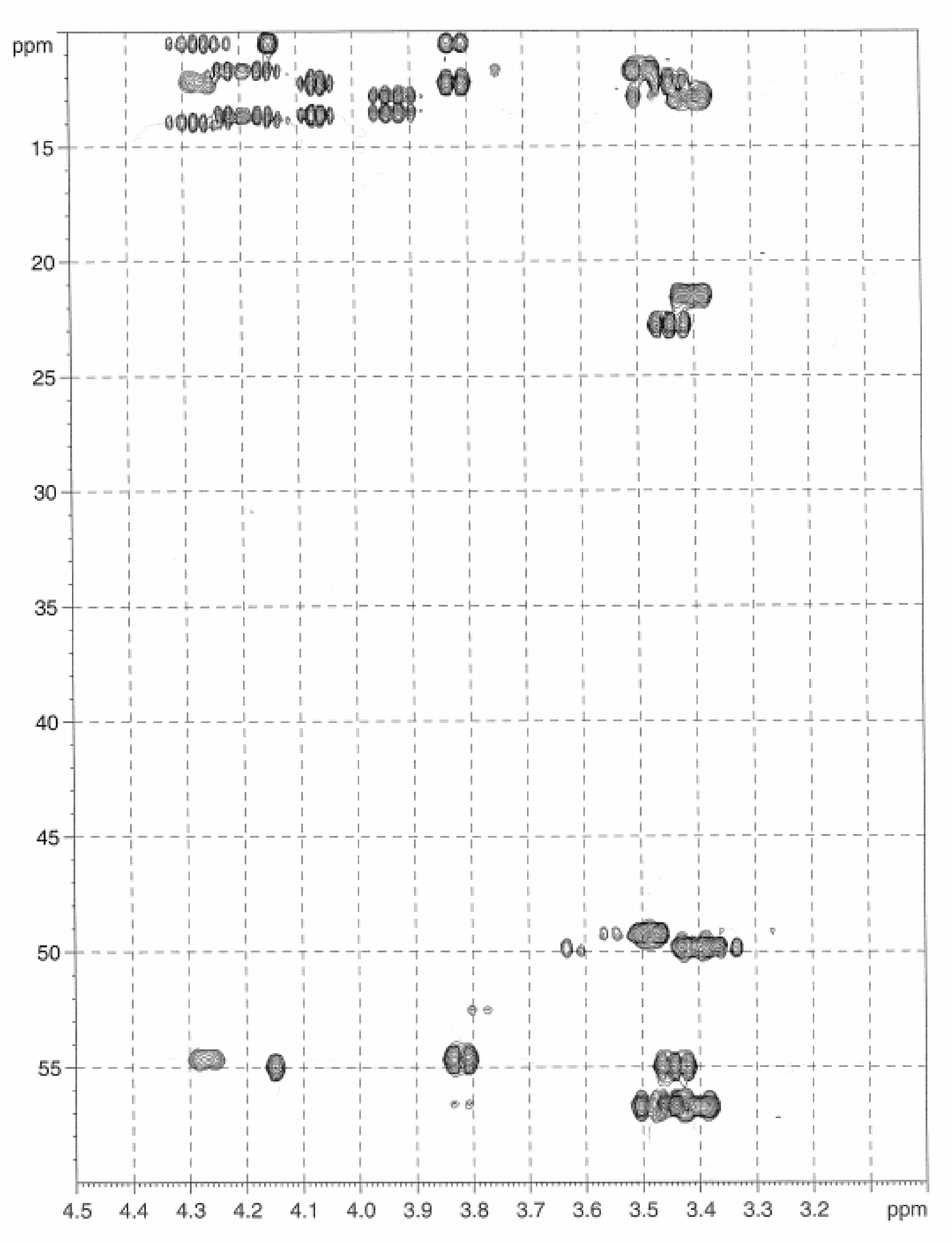

ppm
15
20
25
30
35
40
45
50
55
4.5
4.4
4.3
4.2
4.1
4.0
3.9
3.8
3.7
3.6
3.5
3.4
3.3
3.2
ppm

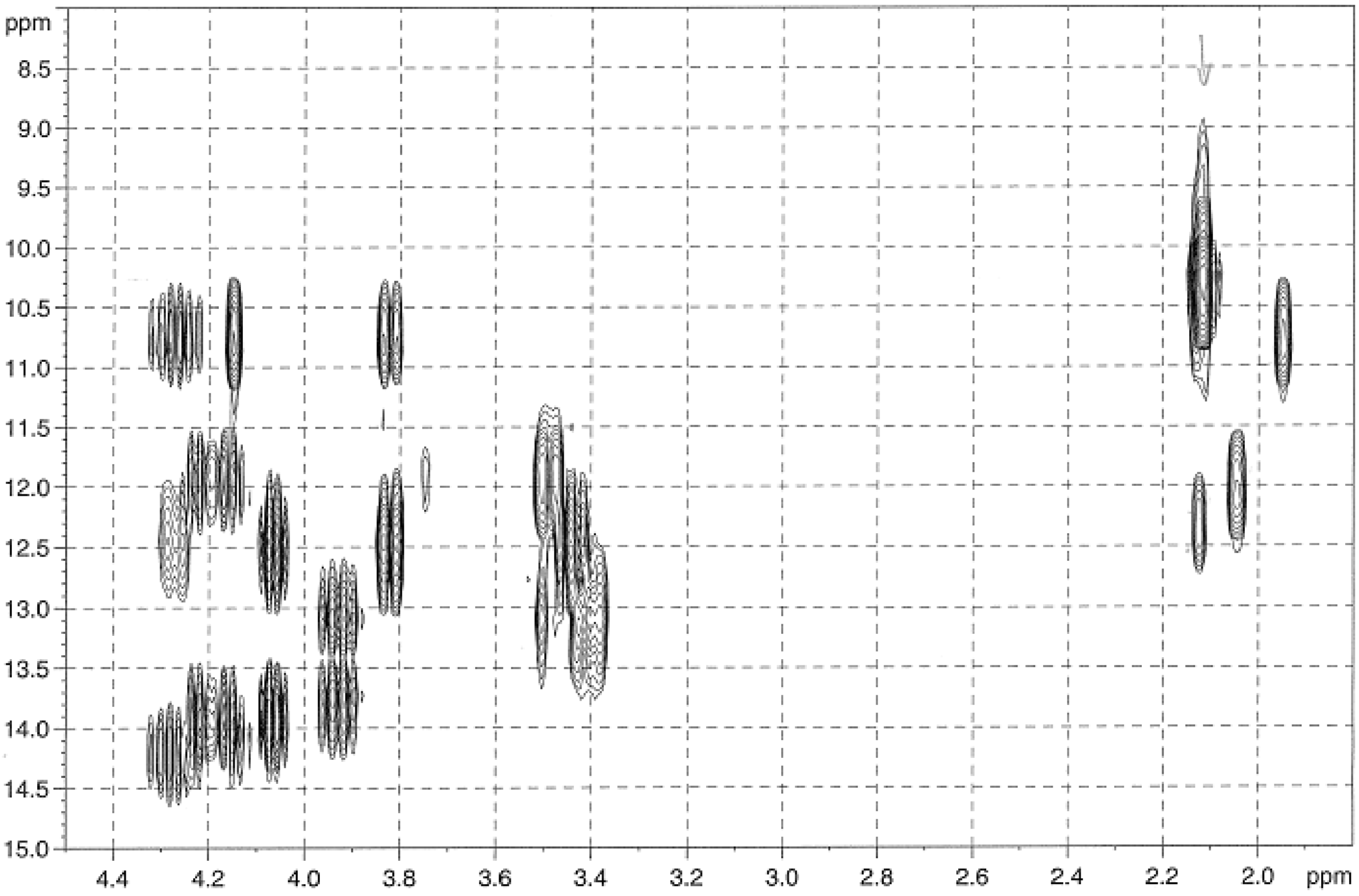

HMBC spectra in $CDCl_3$ of **3** and **5**

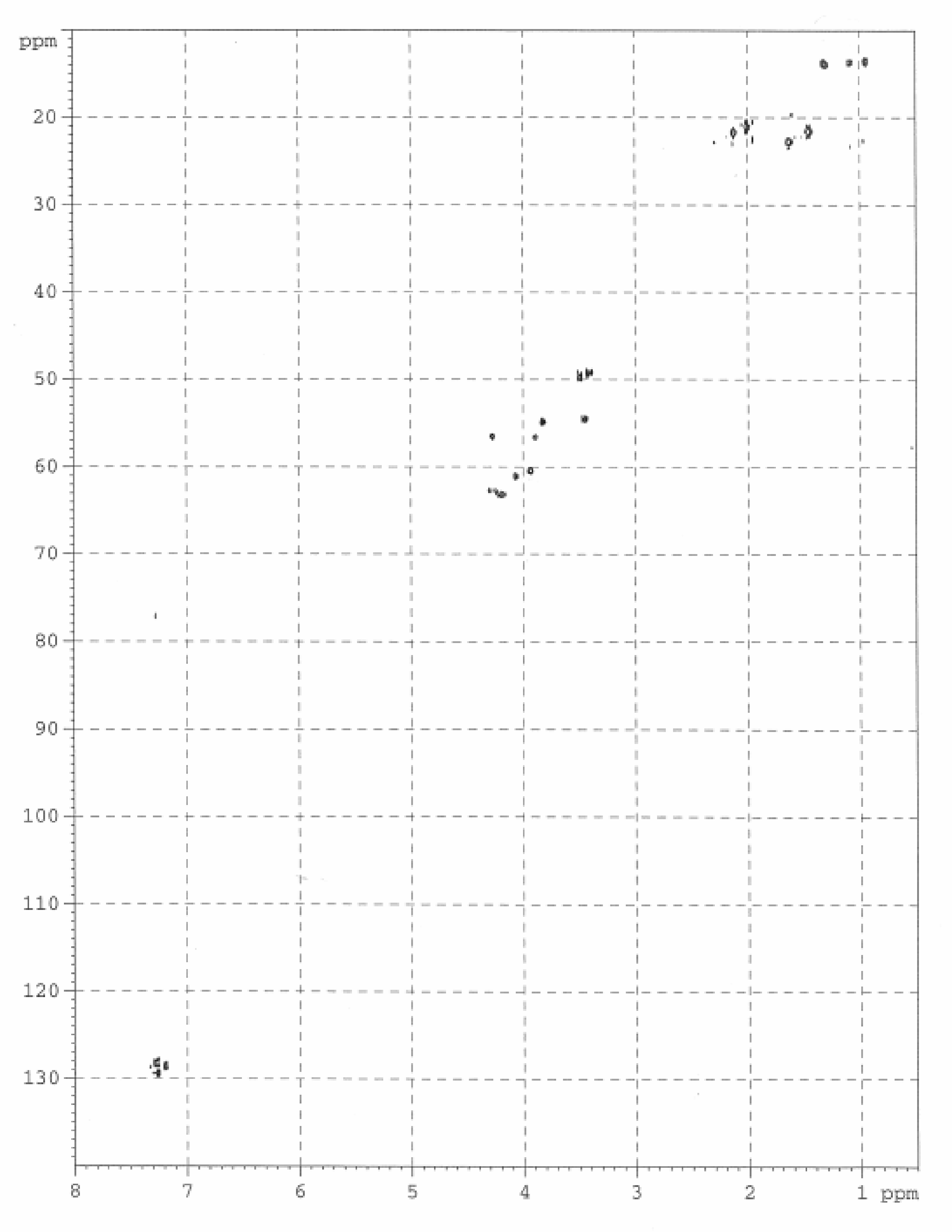

HSQC spectrum in $CDCl_3$ of **3** and **5**

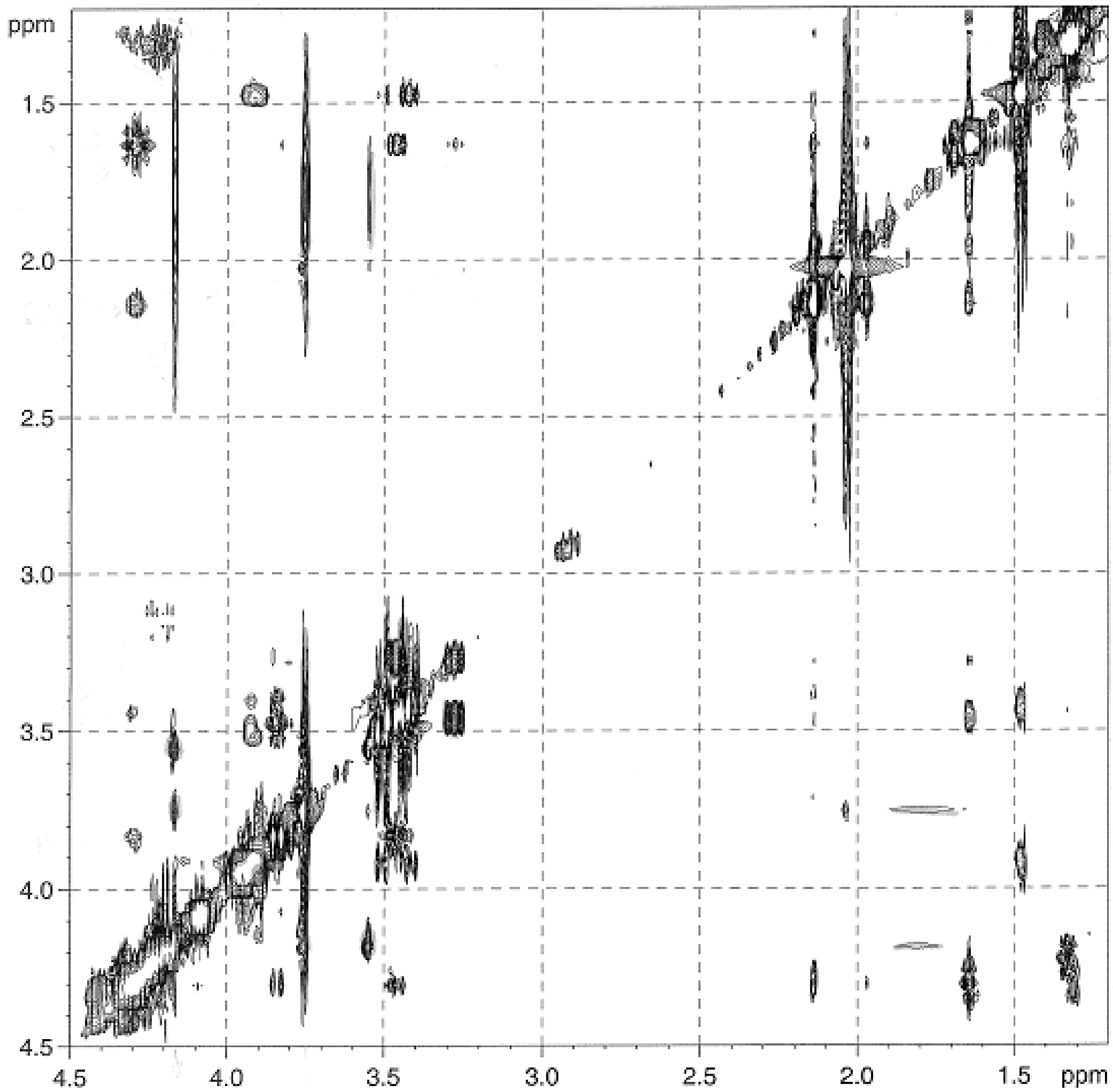

NOESY spectrum in $CDCl_3$ of **3** and **5**

3,5-Diethoxycarbonyl-2,6-dimethyl-3-hydroxy-4-phenyl-1,2-epoxypiperidine (4)

^{1}H NMR (500 MHz, $CDCl_3$, δ): 0.87 (t, *J*= 7.0 Hz, 5-$COOCH_2$*CH_3*, 3H), 1.39 (t, *J* = 7.0 Hz, 3-$COOCH_2$*CH_3*, 3H), 1.45 (s, 2-CH_3, 3H), 1.49 (d, *J* =7.3 Hz, 6-CH_3, 3H), 3.28 (s, 3-OH, 1H), 3.36 (dd, *J*= 13.4 & 10.0 Hz, 5-H, 1H), 3.62 (d, *J*= 13.4 Hz, 4-H, 1H), 3.70 (dq, *J* = 10.0 & 7.3 Hz, 6-H, 1H), 3.86 (AB m, *J* = 7.0 Hz, 5-COO*CH_2*CH_3, 2H), 4.34 (q, *J*= 7.0 Hz, 3-COO*CH_2*CH_3, 2H), 7.12 (d, *J*= 8.0 Hz, ArH, 2H), 7.26 (m, ArH, 3H), 7.25 (m, 5H, H_{arom}) ppm.

^{13}C NMR (125 MHz, $CDCl_3$, δ): 13.5 (5-$COOCH_2$*CH_3*), 13.9 (3-$COOCH_2$*CH_3*), 19.0 (2-CH_3), 20.2 (6-CH_3), 45.4 (4-C), 50.0 (5-C), 56.5 (6-C), 60.5 (5-COO*CH_2*CH_3), 62.9 (3-COO*CH_2*CH_3), 78.4 (3-C), 83.4 (2-C), 127.8 & 128.0 & 128.8 & 134.8 (ArC), 170.5 (5-*CO*OCH_2CH_3), 170.6 (3-*CO*CH_2CH_3) ppm.

IR (dry film, cm^{-1}): 3500 (br), 1740 (br).

Anal. Calcd for $C_{19}H_{25}NO_6$: C, 62.80; H, 6.93; N, 3.85. Found: C, 62.39; H, 6.89; N, 3.74. Mp 76-78 ºC.

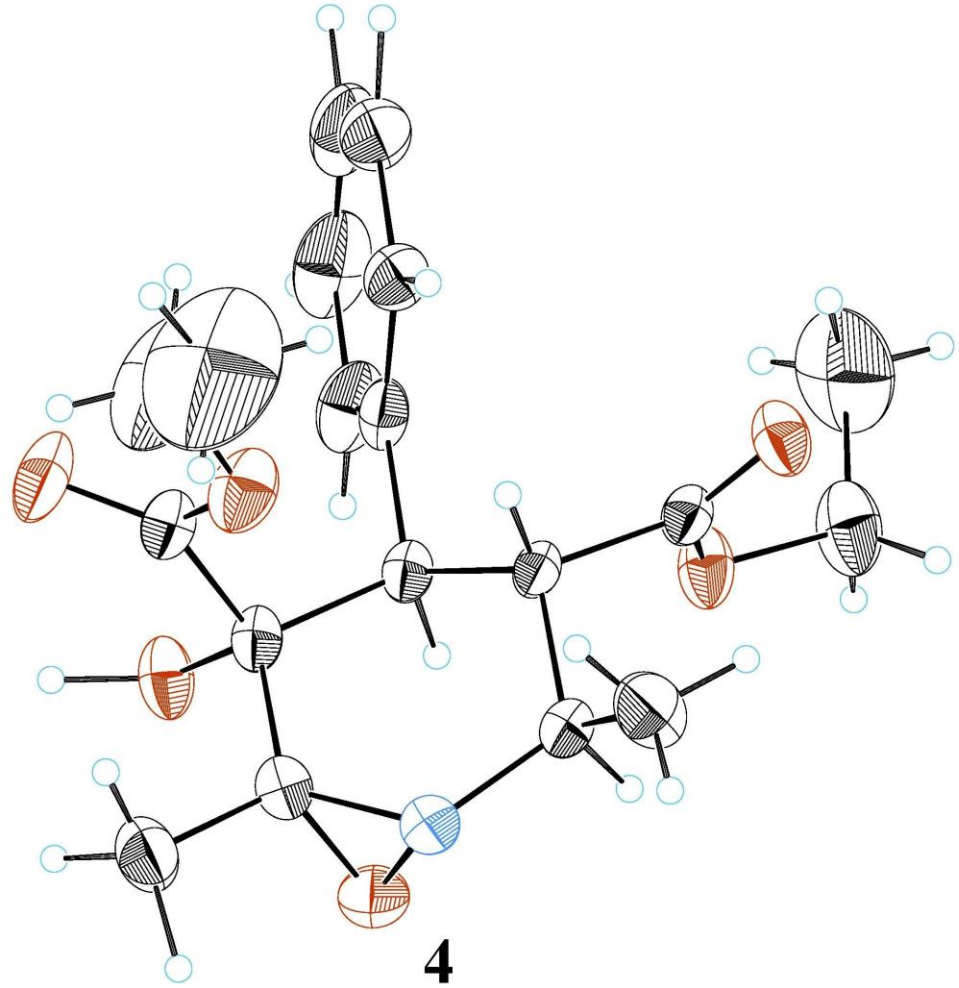

4

X-ray structure (CCDC 667860)

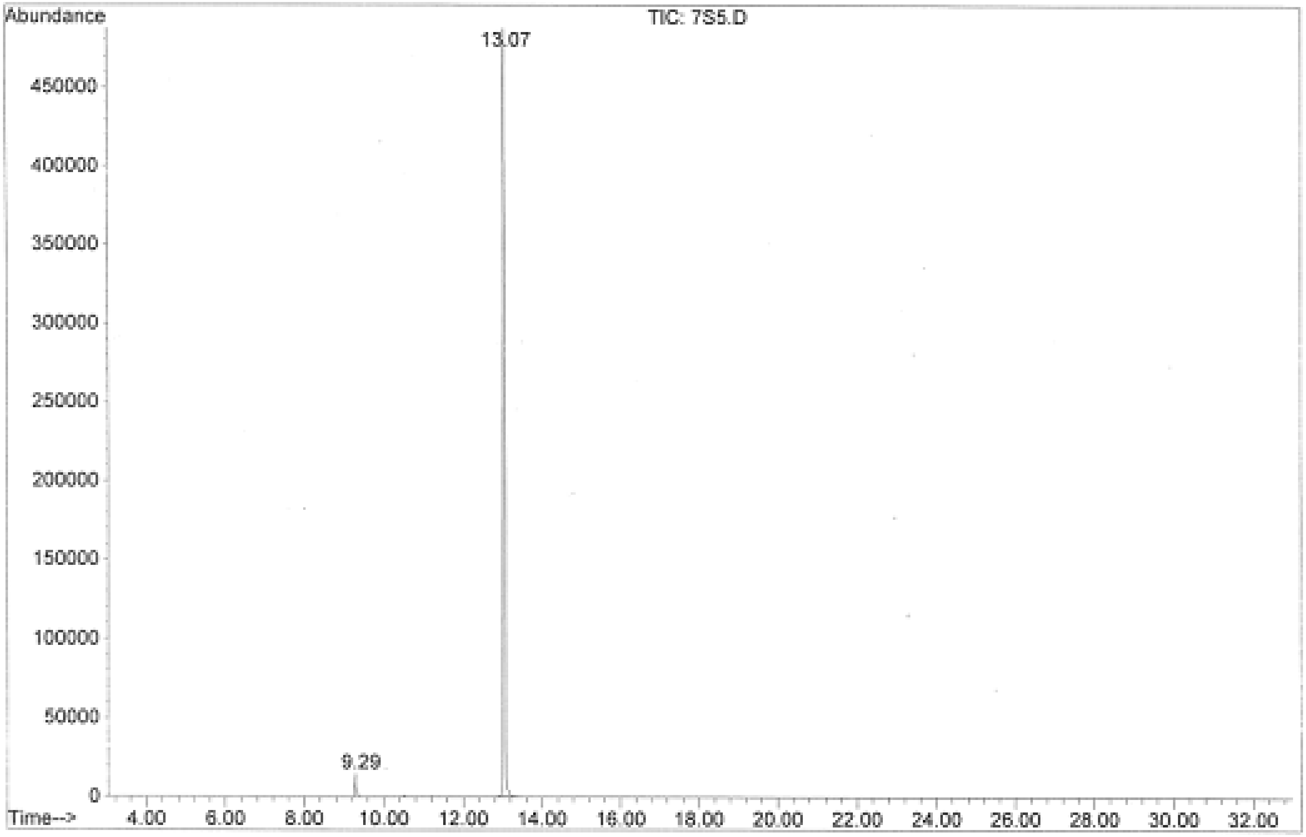

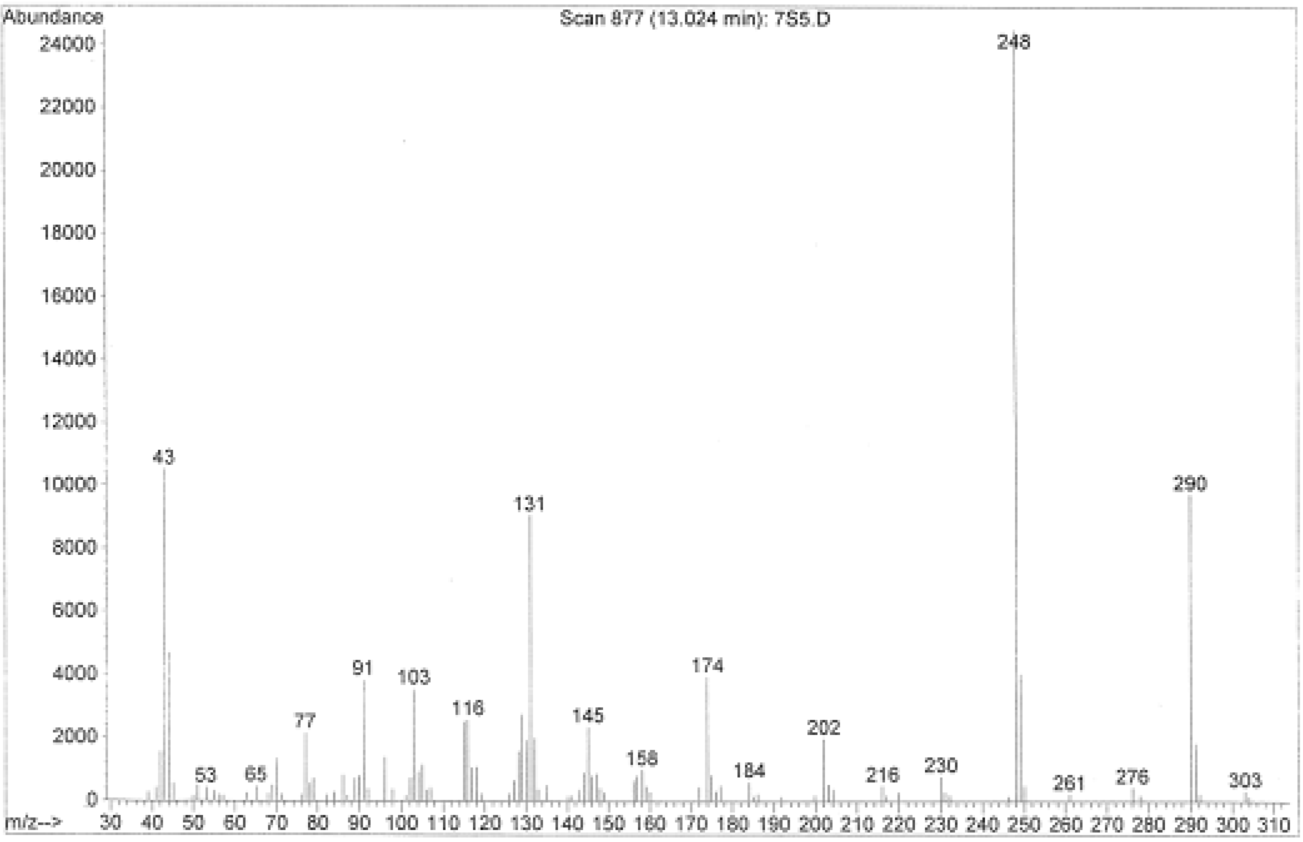

GC–MS spectrum of **4**

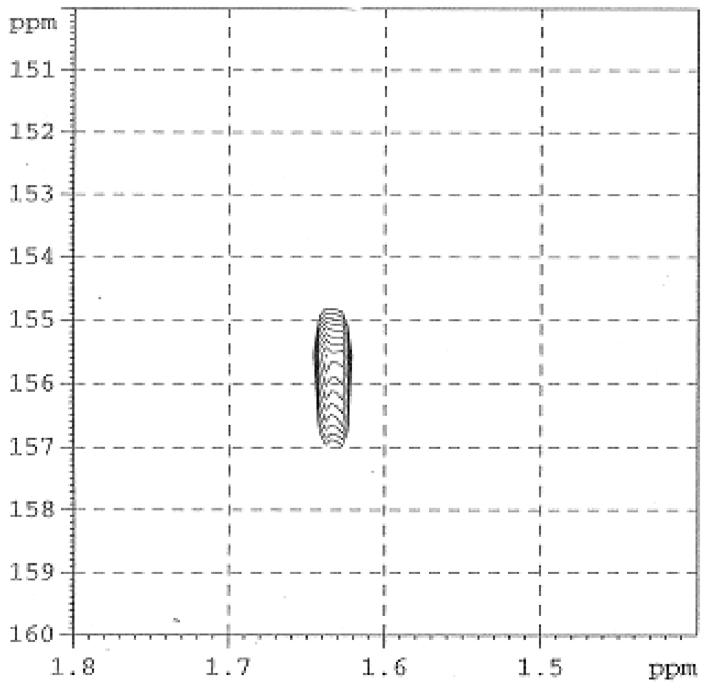

^{15}N NMR spectrum in $CDCl_3$ of **4**

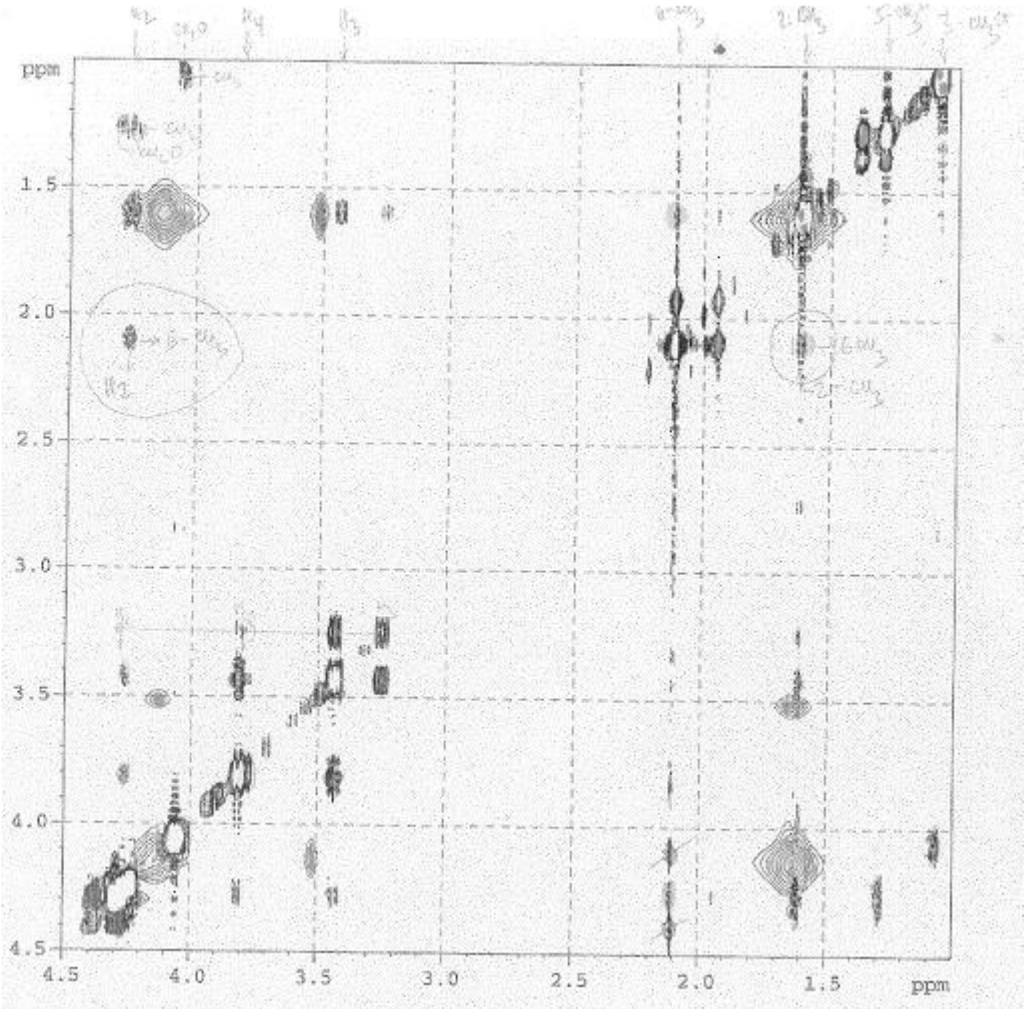

NOESY spectrum in $CDCl_3$ of **4**

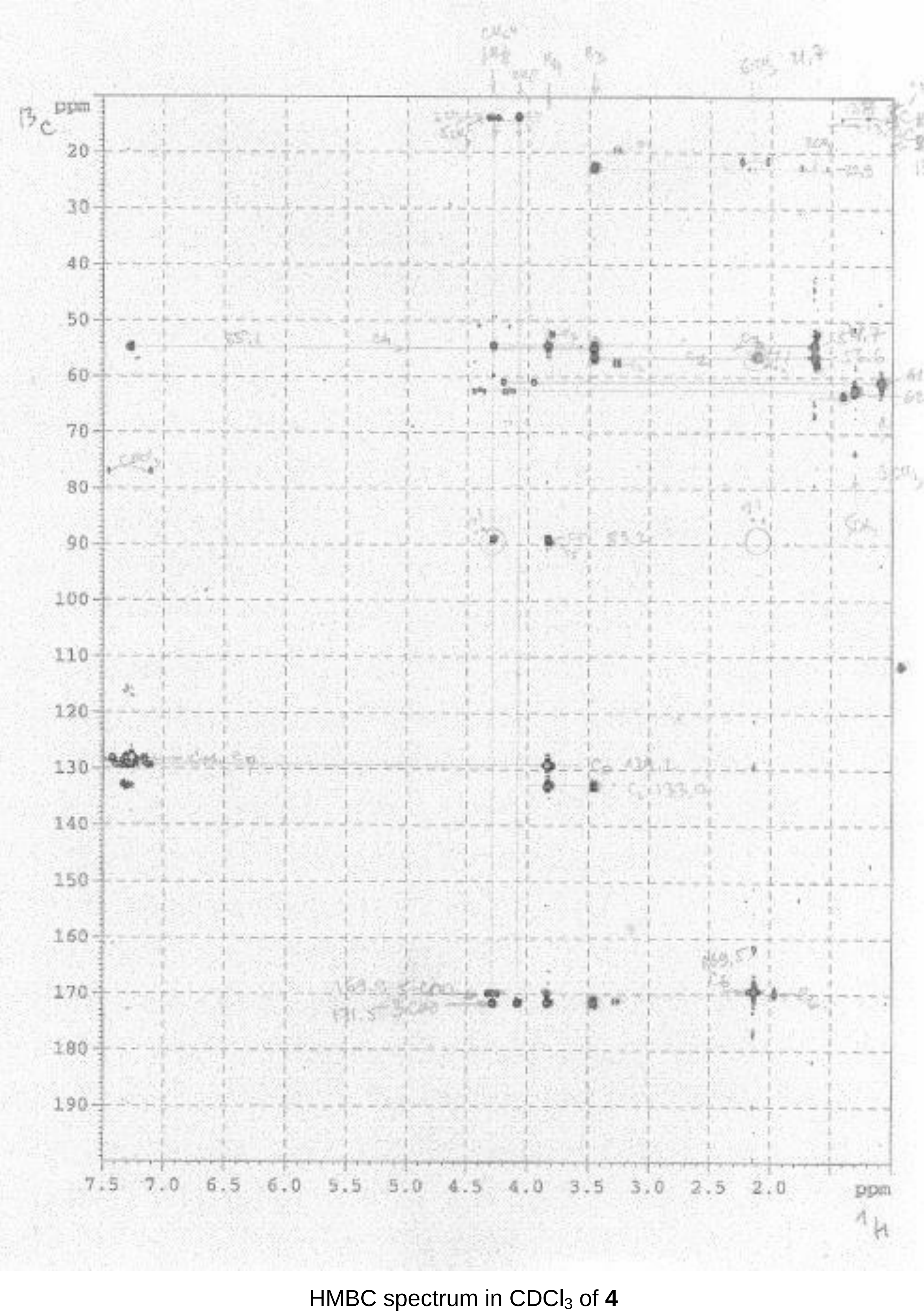

HMBC spectrum in $CDCl_3$ of **4**

N-Acetyl-2,4-diethoxycarbonyl-2-hydroxy-4-methyl-3-phenylpyrolidine (5)

^{1}H NMR (500 MHz, $CDCl_3$, δ): 1.08 (t, *J*= 7.0 Hz, 4-$COOCH_2\mathit{CH_3}$, 3H), 1.33 (t, *J* = 7.0 Hz, 3-$COOCH_2\mathit{CH_3}$, 3H), 1.63 (d, *J*= 6.1 Hz, 5-CH_3, 3H), 2.12 (s, $COCH_3$, 3H), 3.46 (dd, *J*= 12.8 & 7.9 Hz, 4-H, 1H), 3.99 (d, *J*= 12.8 Hz, 3-H, 1H), 4.09 (q, *J*= 7.0 Hz, 4-$COO\mathit{CH_2}CH_3$, 2H), 4.17 (s, OH, 1H), 4.27 (dq, *J* = 7.9 & 6.1 Hz, 5-H, 1H), 4.28 (AB m, *J*= 7.0 Hz, 2-$COO\mathit{CH_2}CH_3$, 2H), 7.20 (m, ArH, 5H) ppm.

^{13}C NMR (125 MHz, $CDCl_3$, δ): 13.81 (4-$COOCH_2\mathit{CH_3}$), 13.90 (2-$COOCH_2\mathit{CH_3}$), 21.03 ($CO\mathit{CH_3}$), 22.94 (5-CH_3), 54.72 (4-C), 55.05 (3-C), 56.62 (5-C), 61.10 (4-$COO\mathit{CH_2}CH_3$), 62.63 (2-$COO\mathit{CH_2}CH_3$), 89.34 (2-C), 129.03 & 129.21 & 132.91 (ArC), 169.10 (*CO*CH_3), 169.62 (2-*CO*OCH_2CH_3), 171.31 (4-*CO*OCH_2CH_3) ppm.

IR (Nujol, cm^{-1}): 3200 (br), 1750 (s), 1725 (s), 1635 (s).

Anal. Calcd for $C_{19}H_{25}NO_6$: C, 62.80; H, 6.93; N, 3.85. Found: C, 62.54; H 6.90; N, 3.72. Mp 82-84 ºC.

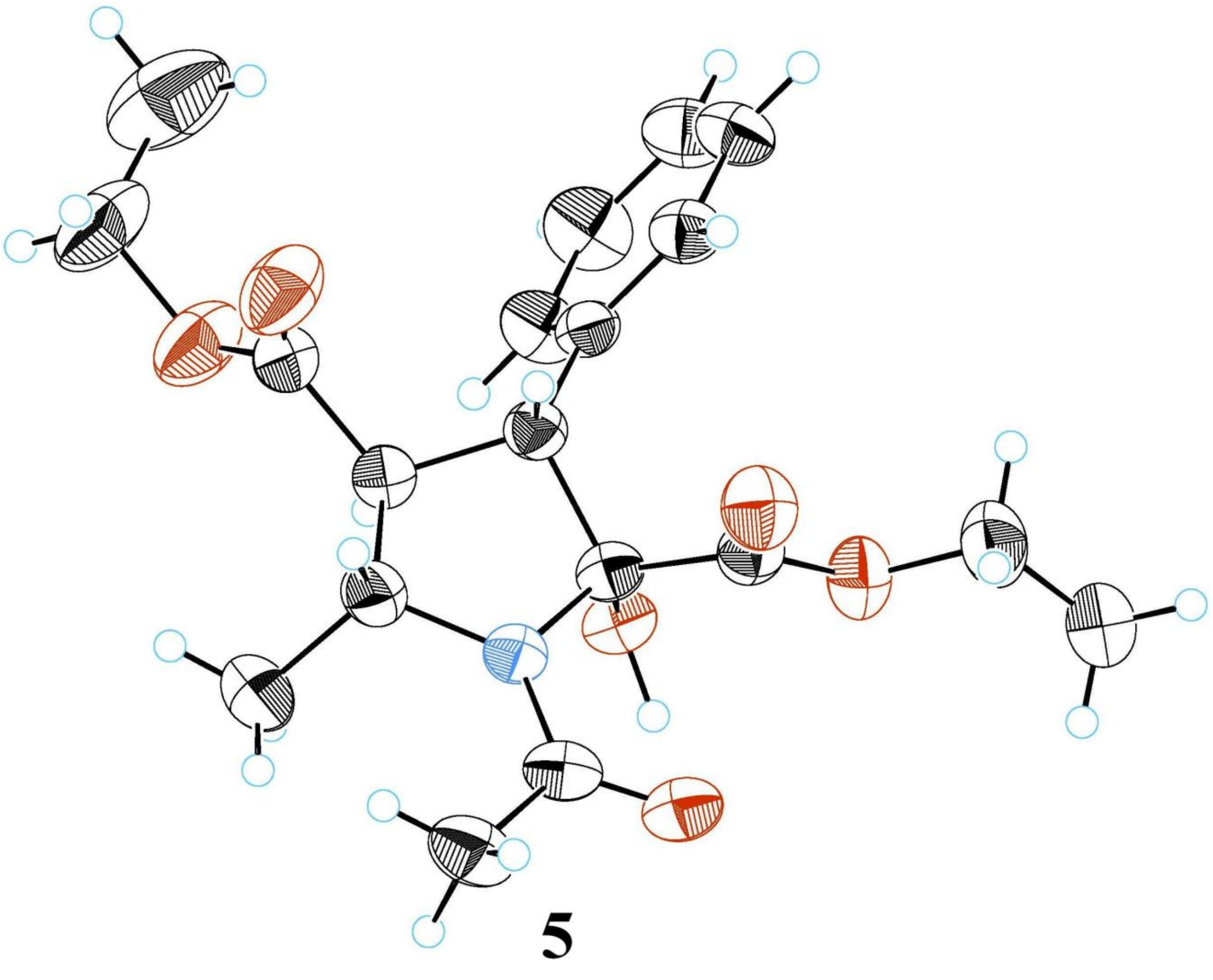

5

X-ray structure (CCDC 1420767)

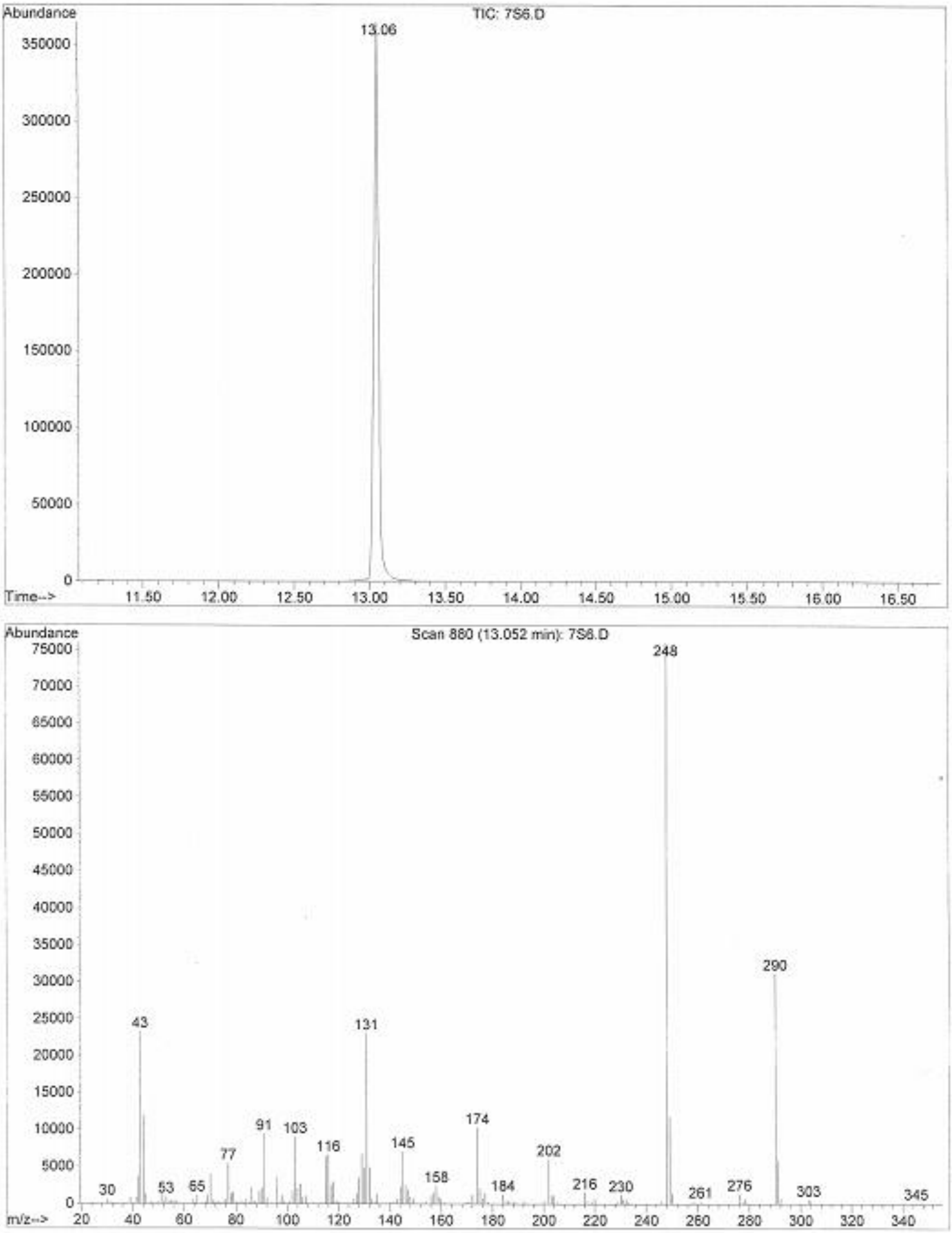

GC–MS spectrum of **5**

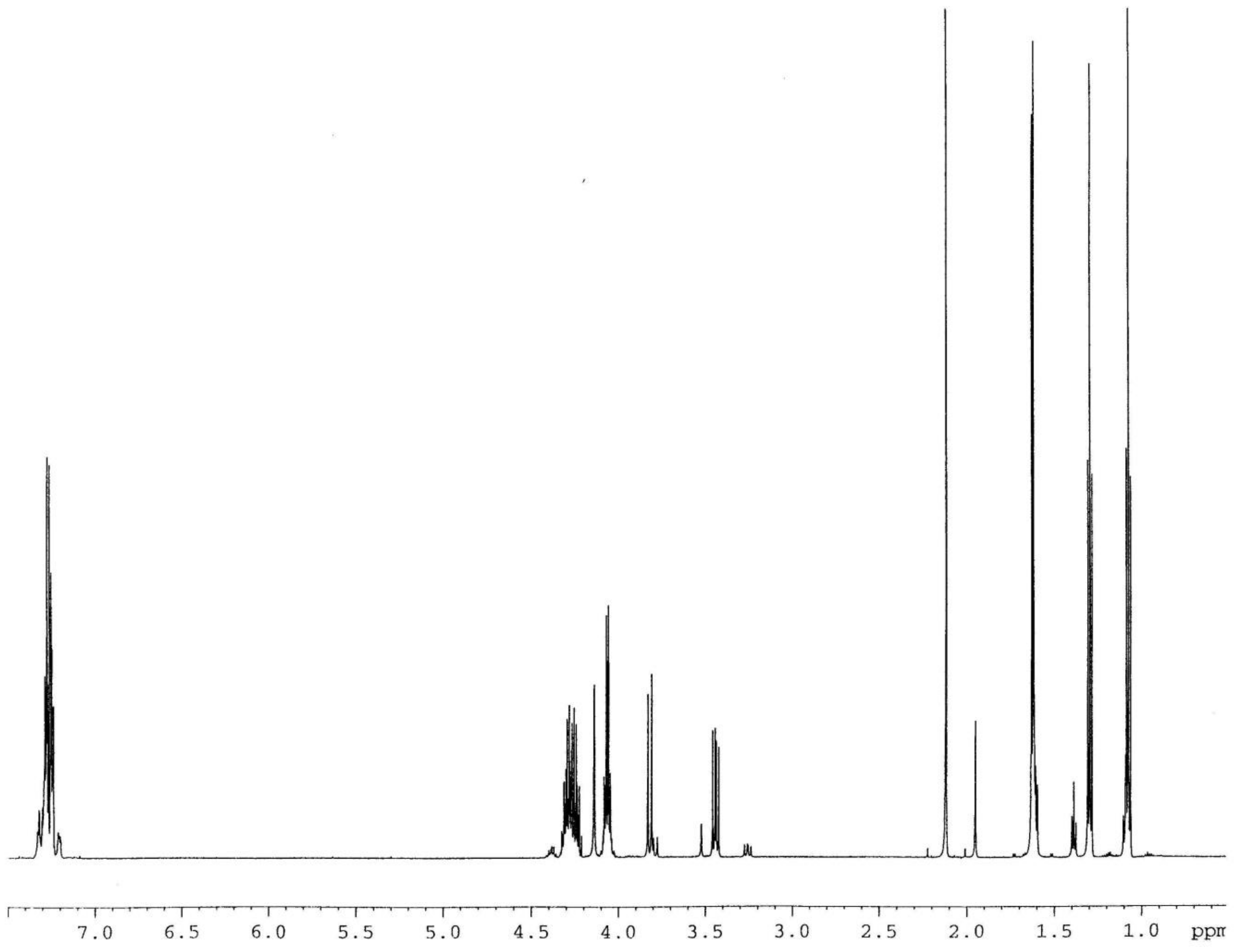
7.0
6.5
6.0
5.5
5.0
4.5
4.0
3.5
3.0
2.5
2.0
1.5
1.0
ppm

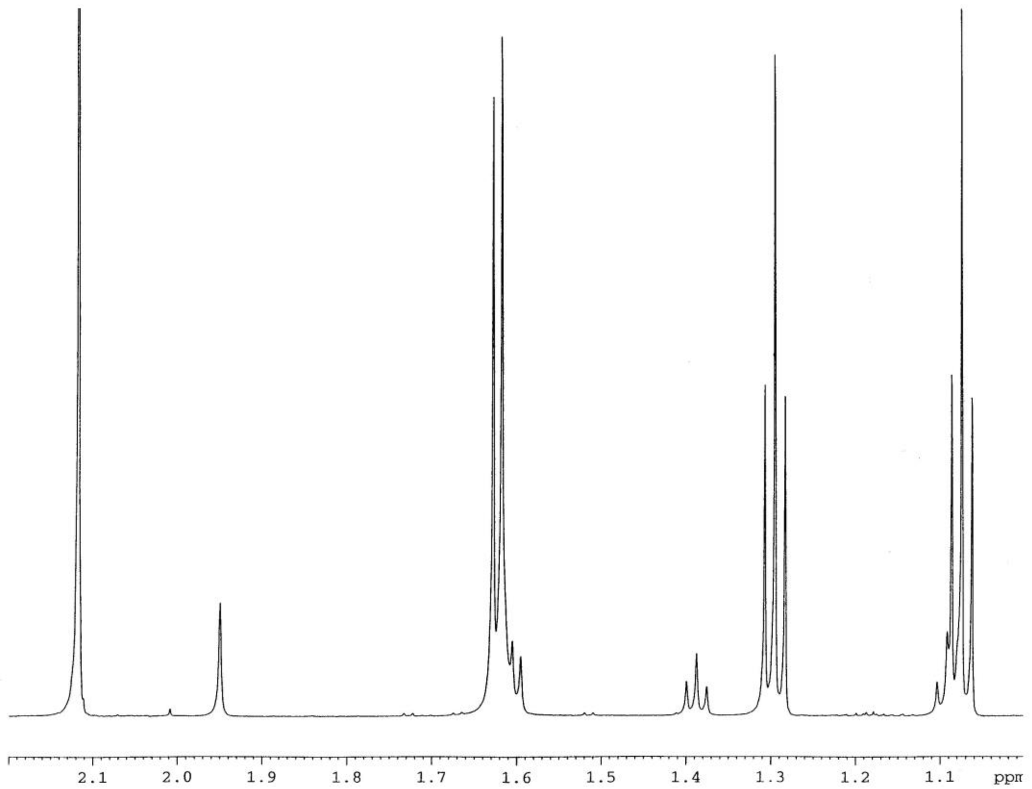
2.1
2.0
1.9
1.8
1.7
1.6
1.5
1.4
1.3
1.2
1.1
ppm

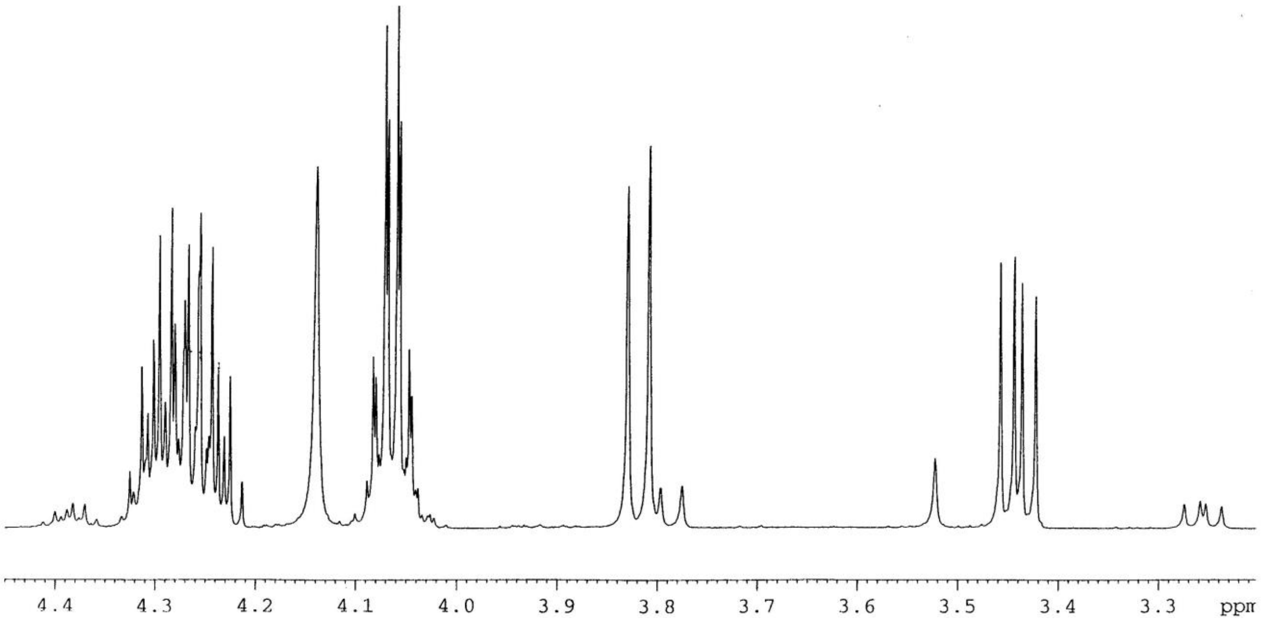

^{1}H NMR spectra in $CDCl_3$ of **5**

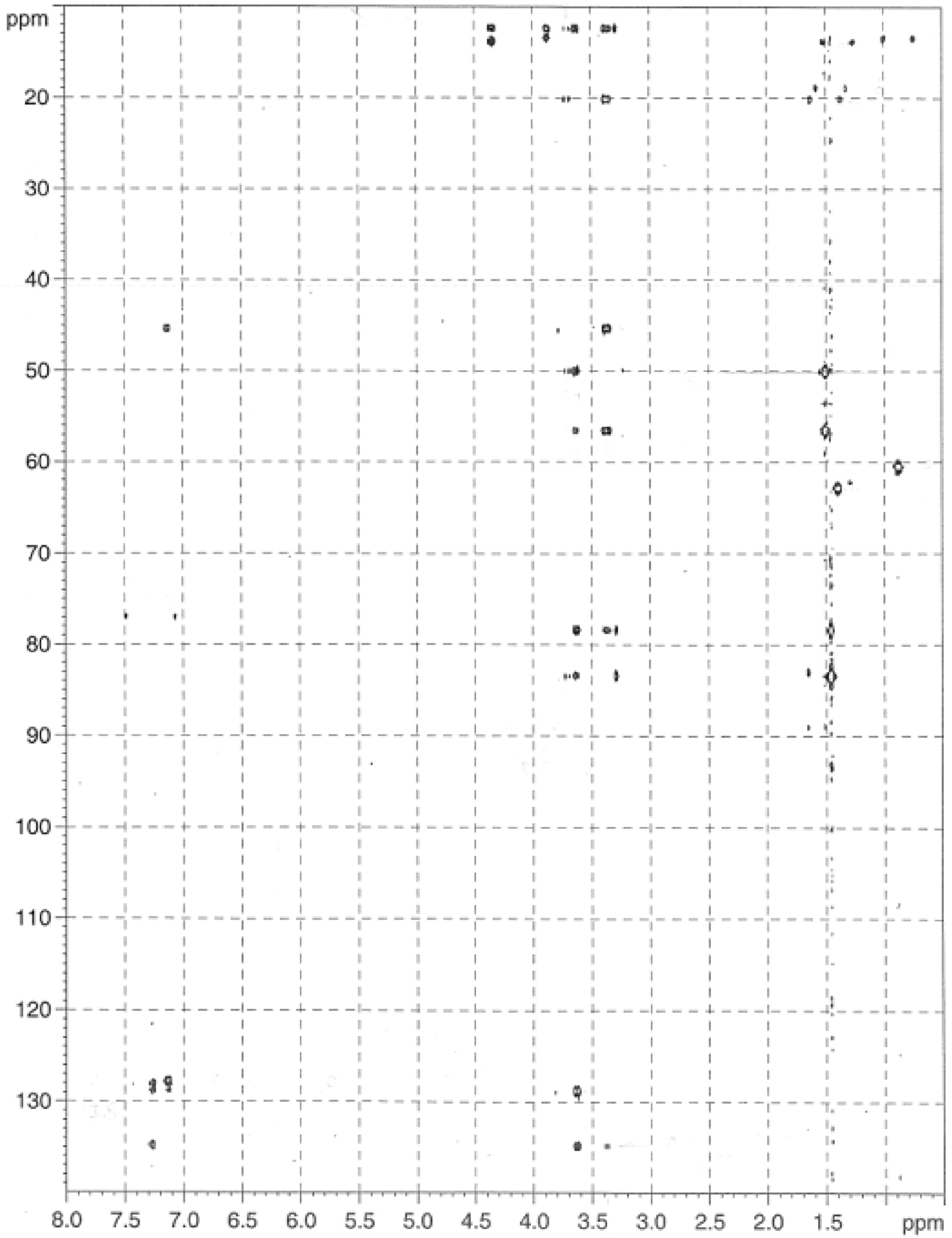

HMBC spectrum in $CDCl_3$ of **5**
